# Nonadditive Transcriptomic Signatures of Genotype-by-Genotype Interactions during the Initiation of Plant-Rhizobium Symbiosis

**DOI:** 10.1128/mSystems.00974-20

**Published:** 2021-01-12

**Authors:** Camilla Fagorzi, Giovanni Bacci, Rui Huang, Lisa Cangioli, Alice Checcucci, Margherita Fini, Elena Perrin, Chiara Natali, George Colin diCenzo, Alessio Mengoni

**Affiliations:** a Department of Biology, University of Florence, Florence, Italy; b Department of Biology, Queen’s University, Kingston, Ontario, Canada; University of Dundee

**Keywords:** RNA-seq, *Sinorhizobium meliloti*, hybrid strain, plant-microbe interactions

## Abstract

Rhizobia are ecologically important, facultative plant-symbiotic microbes. In nature, there is a large variability in the association of rhizobial strains and host plants of the same species. Here, we evaluated whether plant and rhizobial genotypes influence the initial transcriptional response of rhizobium following perception of a host plant. RNA sequencing of the model rhizobium Sinorhizobium meliloti exposed to root exudates or luteolin (an inducer of *nod* genes, involved in the early steps of symbiotic interaction) was performed on a combination of three S. meliloti strains and three alfalfa varieties as host plants. The response to root exudates involved hundreds of changes in the rhizobium transcriptome. Of the differentially expressed genes, 35% were influenced by the strain genotype, 16% were influenced by the plant genotype, and 29% were influenced by strain-by-host plant genotype interactions. We also examined the response of a hybrid S. meliloti strain in which the symbiotic megaplasmid (∼20% of the genome) was mobilized between two of the above-mentioned strains. Dozens of genes were upregulated in the hybrid strain, indicative of nonadditive variation in the transcriptome. In conclusion, this study demonstrated that transcriptional responses of rhizobia upon perception of legumes are influenced by the genotypes of both symbiotic partners and their interaction, suggesting a wide spectrum of genetic determinants involved in the phenotypic variation of plant-rhizobium symbiosis.

**IMPORTANCE** A sustainable way for meeting the need of an increased global food demand should be based on a holobiont perspective, viewing crop plants as intimately associated with their microbiome, which helps improve plant nutrition, tolerance to pests, and adverse climate conditions. However, the genetic repertoire needed for efficient association with plants by the microbial symbionts is still poorly understood. The rhizobia are an exemplary model of facultative plant symbiotic microbes. Here, we evaluated whether genotype-by-genotype interactions could be identified in the initial transcriptional response of rhizobium perception of a host plant. We performed an RNA sequencing study to analyze the transcriptomes of different rhizobial strains elicited by root exudates of three alfalfa varieties as a proxy of an early step of the symbiotic interaction. The results indicated strain- and plant variety-dependent variability in the observed transcriptional changes, providing fundamentally novel insights into the genetic basis of rhizobium-plant interactions. Our results provide genetic insights and perspective to aid in the exploitation of natural rhizobium variation for improvement of legume growth in agricultural ecosystems.

## Abstract (precis)

A sustainable way for meeting the need of an increased global food demand should be based on a holobiont perspective, viewing crop plants as intimately associated with their microbiome, which helps improve plant nutrition, tolerance to pests, and adverse climate conditions. However, the genetic repertoire needed for efficient association with plants by the microbial symbionts is still poorly understood.

## INTRODUCTION

Microbes play a crucial role in the biology and evolution of their eukaryotic hosts (1). Among other activities, microbes contribute to the host’s acquisition of nutrients (2), functioning of the host’s immune system (3), and protection of the host from predation (4). The rules governing host-microbe interactions remain a topic of intense investigation. In many cases, the eukaryotic host selectively recruits the desired microbial partner: squid light organs are selectively colonized by *Vibrio* symbionts (5), legumes select for effective symbionts by sanctioning noneffective symbionts (6), and the crop microbiome is cultivar dependent (7, 8). The genetic basis determining the quality of a microbial symbiont (i.e., its ability to improve host plant phenotypes such as growth and tolerance) and its ability to effectively colonize its eukaryotic partner is generally not well understood, but evolution experiments and high-throughput genome sequencing projects of host-associated microbes and complete microbiomes are shedding light on this topic (9–14). In the case of plants, such studies have observed an enrichment of certain gene functions in plant-associated microbes, such as genes related to carbohydrate metabolism, secretion systems, phytohormone production, and phosphorus solubilization (11, 12, 15, 16).

The rhizobia are an ecologically important exemplar of facultative host-associated microbes. These soil-dwelling bacteria are able to colonize plants and enter an endosymbiotic association with plants of the family *Fabaceae* (17). This developmentally complex process begins with an exchange of signals between the free-living organisms (18), which leads to the invasion of the plant by the rhizobia (19), and culminates in the formation of a new organ (a nodule) in which the plant cells are intracellularly colonized by N_2_-fixing rhizobia (20, 21). Decades of research have identified an intricate network of coordinated gene functions required to establish a successful mutualistic interaction between rhizobia and legumes (21–23). In contrast to the core symbiotic machinery, most of which has been elucidated, much remains unknown about the accessory genes required to optimize the interaction.

In addition to simple gene presence/absence, genotype-by-genotype (GxG) interactions have prominent impacts on symbiotic outcomes (24). The importance of both the plant and bacterial genotypes, and their interaction, in optimizing symbioses between rhizobia and legumes was recognized in early population genetic studies (25–27). More recently, greenhouse studies have directly demonstrated the influence of GxG interactions on the fitness of both the plant and rhizobium partners (28–31). The newly developed select-and-resequence approach is providing a high-throughput approach to uncover the genetic basis underlying GxG interactions for fitness in rhizobium-legume symbioses as well as a way to screen for strain-specific effects of individual genes (32, 33). To date, GxG interaction studies have largely focused on measurements of fitness as a holistic measure of the entire symbiotic process. Nodule formation is a complex developmental process involving several steps, each of which requires a distinct molecular toolkit (34), and in principle, distinct GxG interactions could be acting at each of these developmental stages. Transcriptomic studies have demonstrated that GxG interactions have significant impacts on the gene expression patterns of both partners in mature N_2_-fixing nodules (35, 36). However, we are unaware of studies specifically focusing on the role of GxG interactions in early developmental stages, such as during the initial perception of the partners by each other. Such knowledge is critical not only to fully understand the microevolution of host-associated bacteria but also to develop host variety-specific rhizobium bioinoculants that may ensure good nodulation abilities over unwanted (indigenous) rhizobial strains (37, 38).

Here, we evaluated whether GxG interactions could be identified in the initial transcriptional response of rhizobium perception of a host plant. We worked with Sinorhizobium meliloti, which is one of the best-studied models for GxG interactions in rhizobia. S. meliloti forms N_2_-fixing nodules on plants belonging to the tribe *Trigonelleae* (39), which includes alfalfa, a major forage crop grown worldwide for which many varieties have been developed (40). The S. meliloti genome comprises three main replicons, a chromosome, a chromid, and a megaplasmid; the latter one harbors most of the essential symbiotic functions, including the genes responsible for the initial molecular dialog with the host plant (*nod* genes) (41, 42). To address our aim, the gene expression patterns of three strains of S. meliloti (each with distinct symbiotic properties) following 4 h of exposure to root exudates derived from three alfalfa cultivated varieties were characterized using RNA sequencing (RNA-seq). The transcriptome following exposure to luteolin (a known inducer of *nod* genes, involved in the early steps of symbiotic interaction [43]) was also analyzed. Additionally, the relevance of the megaplasmid in defining the strain-specific transcriptional responses was analyzed by studying a hybrid S. meliloti strain in which the native megaplasmid was replaced with that of another wild-type strain. The results demonstrated that the transcriptional response involved genes on all three replicons and that, even among conserved S. meliloti genes, transcriptional patterns were both strain and root exudate specific.

## RESULTS

### Symbiotic phenotypes differ across rhizobial strain-plant variety combinations.

**FIG 1:**
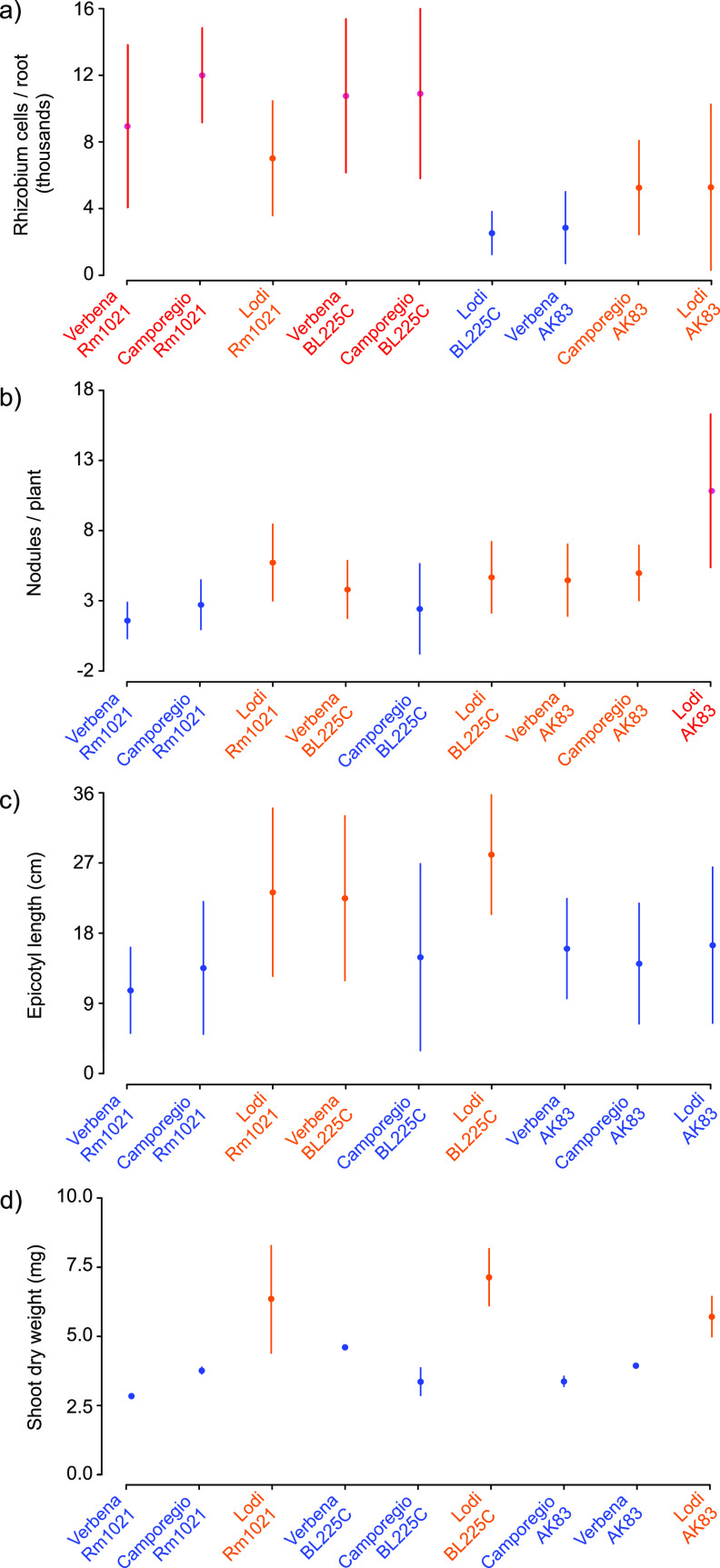
Strain-by-plant variation of symbiosis-associated phenotypes. The number of rhizobium cells retrieved from plant roots (a), number of nodules per plant (b), epicotyl length (c), and plant dry weight (d) are reported. Different colors (pink, orange, and blue) indicate statistically significant groupings (*P* < 0.05) based on a Scott-Knott test. For each condition, the dots indicate the mean values, and the vertical lines indicate the standard deviations.

Symbiotic phenotypes (plant growth and nodule number) and root adhesion of S. meliloti strains Rm1021, BL225C, and AK83 were measured during interactions with three varieties of alfalfa (Camporegio, Verbena, and Lodi). The results indicated that these phenotypes are influenced by both the plant and bacterial genotypes (Fig. 1; see also Fig. S1 in the supplemental material). Root adhesion phenotypes (Fig. 1a) were divided by the Scott-Knott test into three main groups reflecting high, medium, and low root colonization. Interestingly, each group was heterogeneous with respect to both plant variety and S. meliloti strain, consistent with the specificity of plant variety (i.e., genotype *sensu lato*) and strain individuality (i.e., strain genotype) pairs in root colonization efficiency. For instance, S. meliloti BL225C strongly colonized the roots of the Camporegio and Verbena varieties, but it displayed much weaker colonization of the Lodi cultivar. On the other hand, S. meliloti AK83 colonized the Lodi and Camporegio varieties better than the Verbena cultivar. Nodules per plant as well as measures of symbiotic efficiency (epicotyl length and shoot dry weight) showed differences among the strain-variety combinations (Fig. 1b to d). However, the extents of the variation were lower than those recorded for plant root adhesion. The highest number of nodules was found on the Lodi variety nodulated by S. meliloti AK83, which was previously interpreted as a consequence of its reduced N_2_ fixation ability with some alfalfa varieties (44–46). Interestingly, the measures of symbiotic efficiency did not correlate with root adhesion phenotypes (both adhesion versus dry weight and adhesion versus epicotyl length gave nonsignificant Pearson correlation values [*P* > 0.18]). However, we cannot *a priori* exclude that measuring adhesion over the whole root might not reflect adhesion to the root hair extension zone, where rhizobia start the symbiotic interaction. For example, the largest plants were the Lodi variety inoculated with S. meliloti BL225C despite the root adhesion of this combination being the lowest. Similarly, the smallest plants were the Verbena variety inoculated with S. meliloti Rm1021 despite strong root adhesion in this pairing.

10.1128/mSystems.00974-20.2 FIG S1 Symbiosis-associated phenotypes. The number of rhizobium cells retrieved from plant roots (a), number of nodules per plant (b), epicotyl length (c), and the plant dry weight (d) are reported. Letters and different colors indicate groupings based on Tukey contrasts (*P* < 0.05). Error bars indicate 1 standard deviation. Download FIG S1, PDF file, 0.04 MB. Copyright © 2021 Fagorzi et al. 2021 Fagorzi et al. This content is distributed under the terms of the Creative Commons Attribution 4.0 International license.

### Root exudates differ among alfalfa varieties.

**FIG 2:**
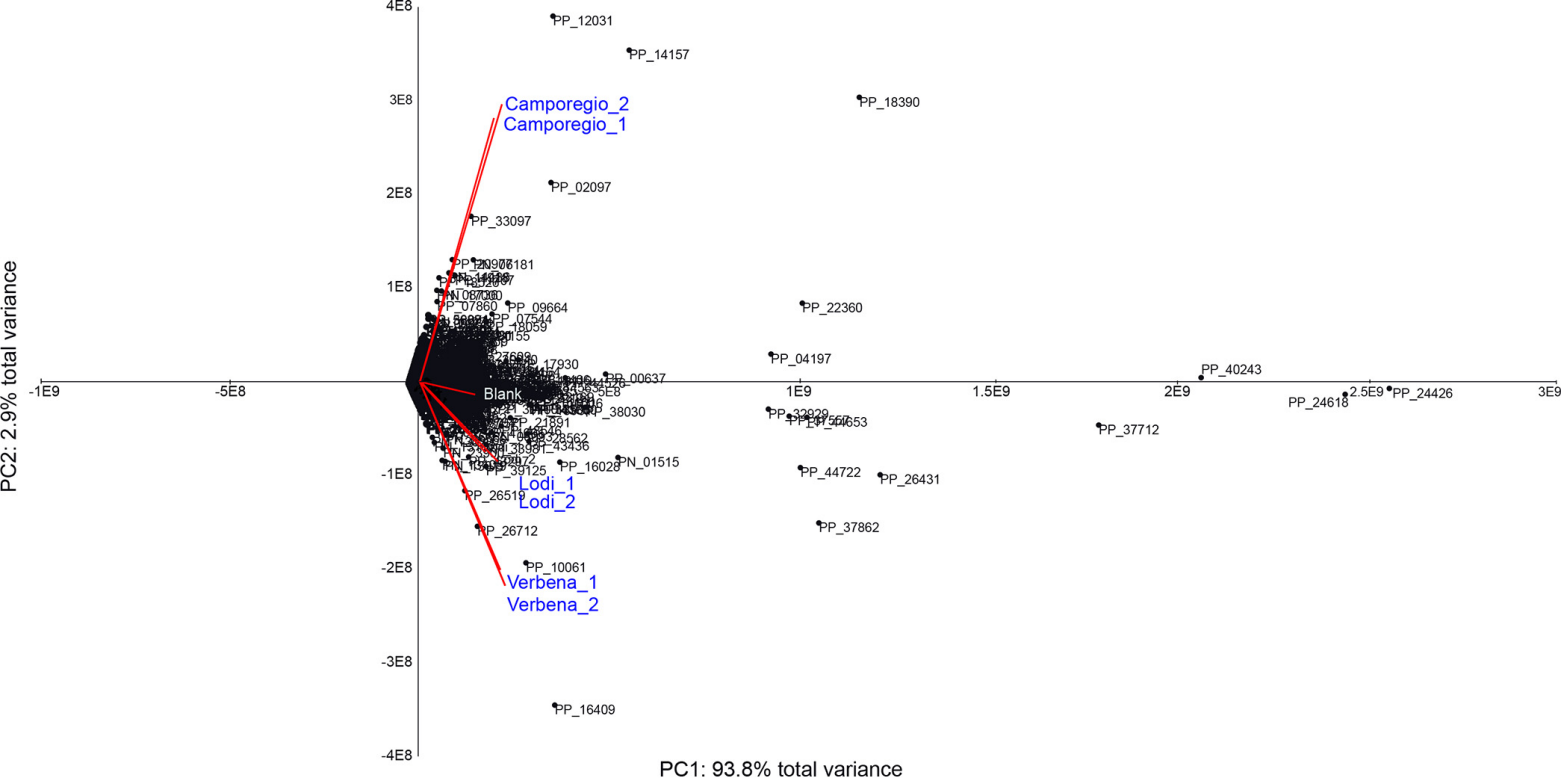
Principal-component analysis biplot from LC-MS analysis of root exudates of the Verbena, Lodi, and Camporegio varieties of alfalfa, including the blank control. Centroids report LC-MS peak IDs (see worksheet 1 in Data Set S1 in the supplemental material), and vectors indicate the loadings of plant varieties.

Liquid chromatography-mass spectrometry (LC-MS) analysis of the alfalfa root exudates detected a total of 2,688 unique features, including 392 annotated features, across the two platforms: 1,514 hydrophilic features were detected by ultraperformance liquid chromatography (UPLC)-MS in positive mode (PP) (288 annotated), and 1,174 hydrophilic features were detected by UPLC-MS in negative mode (PN) (104 annotated) (see worksheet 1 in Data Set S1 in the supplemental material). In order to clarify if the metabolite compositions of the root exudates from the alfalfa varieties differed, principal-component analysis (PCA) was performed on the two biological replicates of the three cultivars (Fig. 2). The three cultivars clearly grouped separately from each other, suggesting the presence of variety-specific differences in their metabolic compositions. Peaks PP_23583, PP_25608, PP_14051, and PP_23300 were assigned by the PubChem database to liquiritigenin, apigenin, genistein, and apigeninidin, respectively; however, differences in the concentrations of these compounds between the root exudates were not statistically significant (data not shown). Most of the observed differences were related to amino acids, in particular *N*-acetyl-l-leucine, tryptophan, cytosine, 3,5-dihydroxyphenylglycine, and the dipeptide Val-Ala (Table S1). Multiple flavones and flavonoids, which include known inducers of NodD activation (43) and chemotaxis (47), were potentially identified. These include a peak hypothetically attributed to apigeninidin (PP_23300), which was found in the Verbena and Camporegio root exudates; liquiritigenin (PP_23583), which was found in the Camporegio and Lodi root exudates; as well as apigenin (PP_25608) and genistein (PP_14051), which were found in variable amounts in the root exudates from all three varieties. Elemental analysis (CHNS [carbon, hydrogen, nitrogen, sulfur]) of root exudates was also performed (Table S2), and the results were used to normalize the quantity of root exudates used in the treatment of S. meliloti strains based on equalizing the amount of total organic carbon (TOC) added to each culture.

10.1128/mSystems.00974-20.6 TABLE S1 Metabolites with the greatest differences among root exudates. Results of Simper analysis are based on the decomposition of the Bray-Curtis dissimilarity obtained from each peak ID value. The peak ID, average dissimilarity, percent contribution, and hypothetical compound after a search of the PubChem library (https://pubchem.ncbi.nlm.nih.gov) are reported. Download Table S1, PDF file, 0.02 MB. Copyright © 2021 Fagorzi et al. 2021 Fagorzi et al. This content is distributed under the terms of the Creative Commons Attribution 4.0 International license.

10.1128/mSystems.00974-20.7 TABLE S2 Chemical composition of root exudates. The percentages of nitrogen (N), carbon (C), and hydrogen (H) are reported for the root exudates of the three cultivars and the blank (medium stored in the apparatus used to collect root exudates). n.d., not detected (detection limit of 0.001% for each considered element). Sulfur was not in any sample and is omitted from the table. Download Table S2, PDF file, 0.01 MB. Copyright © 2021 Fagorzi et al. 2021 Fagorzi et al. This content is distributed under the terms of the Creative Commons Attribution 4.0 International license.

10.1128/mSystems.00974-20.10 DATA SET S1 Details on LC-MS data and differentially expressed genes. (Worksheet 1) List of LC-MS peaks; (worksheet 2) significantly differentially expressed genes (DEGs); (worksheet 3) overall number of DEGs in the strains by treatment combinations, their location on the S. meliloti replicons, and up- and downregulation with respect to the blank control; (worksheet 4) pangenome ortholog assignments from Roary. Download Data Set S1, XLSX file, 0.8 MB. Copyright © 2021 Fagorzi et al. 2021 Fagorzi et al. This content is distributed under the terms of the Creative Commons Attribution 4.0 International license.

### The number of differentially expressed genes changes in strain-condition combinations.

The global transcriptional responses of the three S. meliloti wild-type strains following a 4-h exposure to luteolin (the model flavone involved in the early steps of symbiotic interaction [43]) or alfalfa root exudates were evaluated using RNA sequencing. In addition, a fourth strain (BM806, referred to as “hybrid” for simplicity) was included (48); the results for this strain are discussed below. A list of differentially expressed genes (DEGs) for each strain and condition (luteolin and the root exudates of the three plant varieties) against the control (blank sample) is reported in Data Set S1, worksheet 2. DEGs were considered to be biologically significant if they had a ≥2-fold change in expression and an adjusted *P* value of <0.01. The numbers of DEGs are shown in Table 1 (also see Data Set S1, worksheet 3). Reverse transcriptase quantitative PCR (RT-qPCR) on a panel of seven DEGs validated the reliability of the RNA-seq data (Table S3).

**TABLE 1:** Significant DEGs^*a*^

Strain	No. of significant DEGs (%)			
	Camporegio	Lodi	Verbena	Luteolin
1021	516 (8.79)	32 (0.55)	506 (8.62)	36 (0.61)
AK83	357 (5.84)	66 (1.08)	192 (3.14)	60 (0.98)
BL225C	1,159 (19.33)	76 (1.27)	693 (11.56)	149 (2.49)
Hybrid	503 (8.38)	98 (1.63)	325 (5.41)	52 (0.87)

a The number of significant DEGs with respect to the blank control (2-fold change in expression and an adjusted *P* value of ≤0.01) and the percentage with respect to the total number of genes are reported.

10.1128/mSystems.00974-20.8 TABLE S3 Results of quantitative RT-PCR on selected genes. Download Table S3, CSV file, 0.01 MB. Copyright © 2021 Fagorzi et al. 2021 Fagorzi et al. This content is distributed under the terms of the Creative Commons Attribution 4.0 International license.

**FIG 3:**
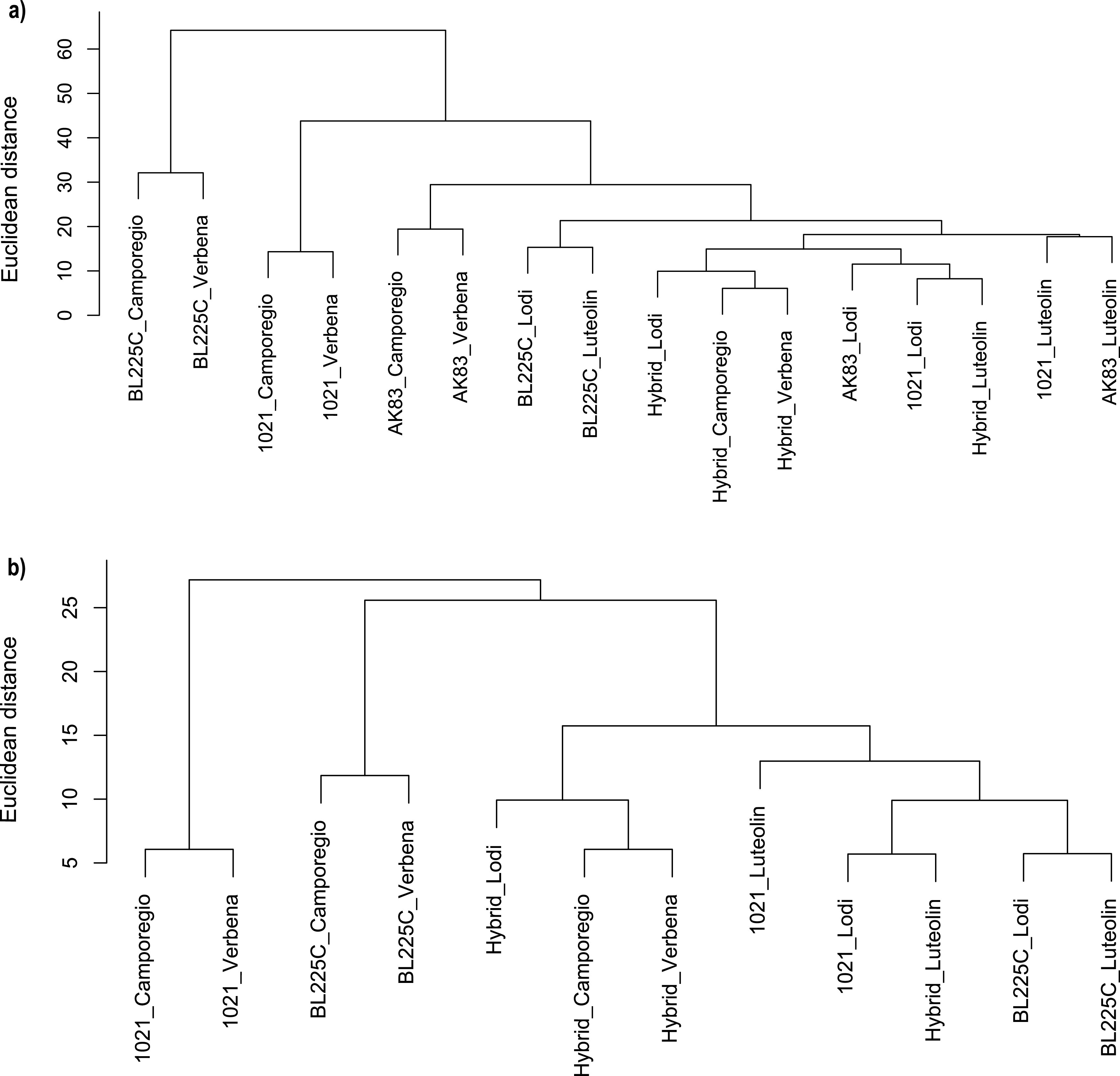
Cluster analyses of the expression profiles of the conserved gene sets of strains grown in the presence of the three root exudates and luteolin. (a) Full data set of DEGs identified in the four strains. (b) DEGs of orthologs of pSymA-like only. Hierarchical clustering was performed in R with the hclust function based on Euclidean distance, with the “complete” agglomeration method. Bars represent Euclidean distance.

In general, luteolin treatment resulted in the lowest number of DEGs, ranging from 36 to 149 per strain. Concerning the root exudates, the number of DEGs was influenced by both the strain and the alfalfa cultivar. Overall, the Camporegio and Verbena root exudates induced more gene expression changes than the Lodi root exudate. Cluster analyses of all genes that were differentially expressed under at least one condition (fold change of ≥2; adjusted *P* value of <0.01) revealed that for each strain, the transcriptional responses to the Verbena and Camporegio root exudates were similar and grouped separately from that of the Lodi cultivar (Fig. 3a; Fig. S2 [see also supplemental File S1 at https://doi.org/10.5061/dryad.jdfn2z38q]). Interestingly, ∼80% of the genes upregulated by root exudates were found on the chromosomes of the three strains, whereas ∼77% of the downregulated genes were found on the pSymA and pSymB replicons (Data Set S1, worksheet 3). This is consistent with a previous signature-tagged mutagenesis study reporting that 80% of genes required for rhizosphere colonization are chromosomally located in S. meliloti Rm1021 (49).

10.1128/mSystems.00974-20.3 FIG S2 Cluster analysis of the stimulons for the four strains. Differentially expressed genes under each condition are on the columns. (a) Rm1021; (b) BL225C; (c) AK83; (d) hybrid strain. Download FIG S2, PDF file, 0.2 MB. Copyright © 2021 Fagorzi et al. 2021 Fagorzi et al. This content is distributed under the terms of the Creative Commons Attribution 4.0 International license.

**FIG 4:**
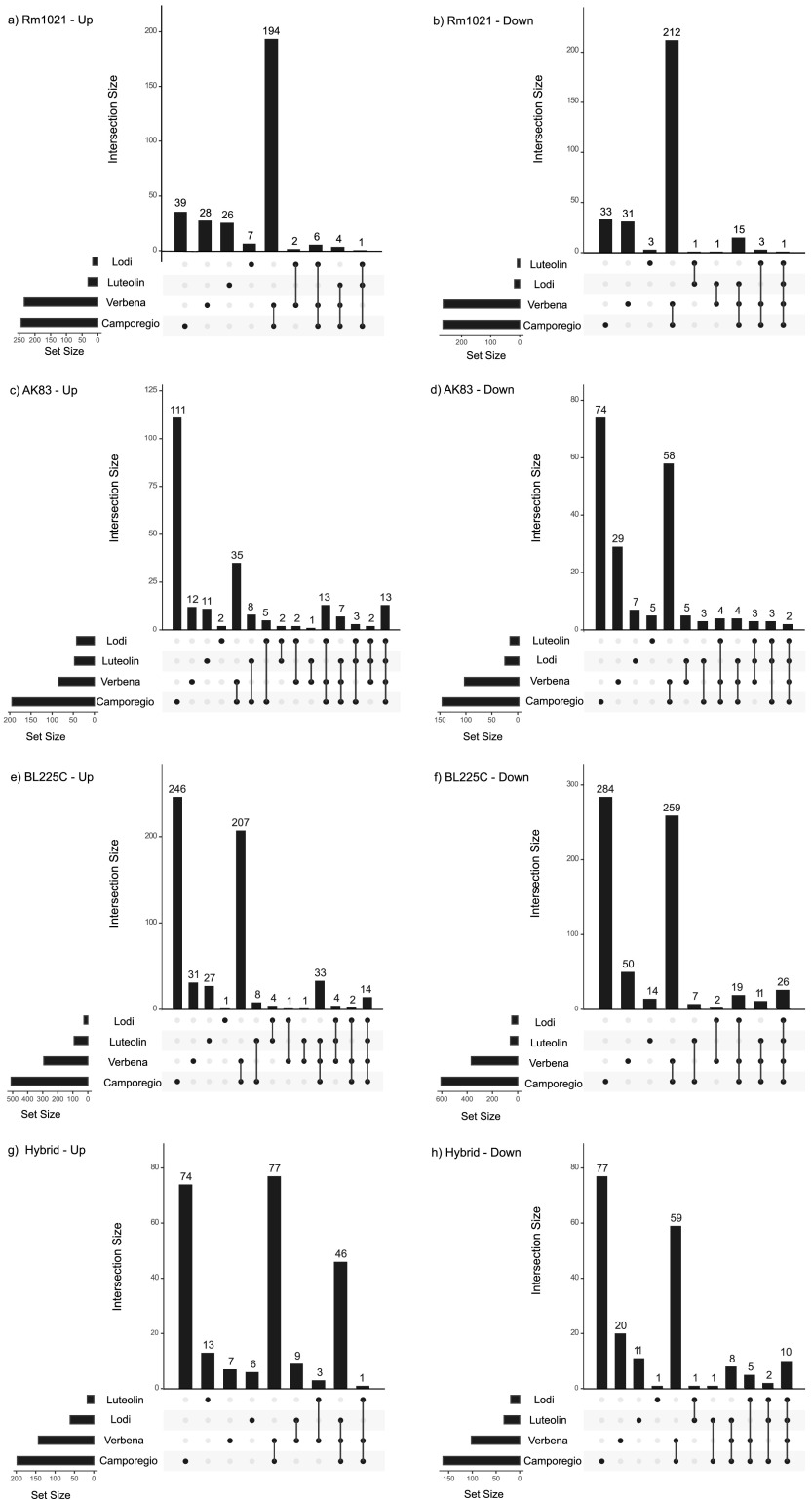
Intersection between up- and downregulated genes under all conditions. The plot reports the numbers of upregulated (a, c, and e) and downregulated (b, d, and f) genes under each condition for each strain. (a and b) Rm1021; (c and d) BL225; (e and f) AK83; (g and h) hybrid strain. Each row of the matrix corresponds to a condition, with the size (number of upregulated/downregulated genes) reported to the left as a bar plot. Each column corresponds to one intersection (similar to a Venn diagram): cells are either empty, indicating that up- or downregulated genes under the specified conditions are not part of the intersection, or filled, indicating that the genes present under the specified conditions are participating at the intersection. Bars on the top show the size of the intersection reported on the bottom.

Under all conditions, S. meliloti BL225C displayed the largest number of DEGs (with up to 20% of genes differentially expressed) (Fig. 4e and f), while S. meliloti AK83 had the fewest (Fig. 4c and d). The majority of DEGs (>75%) had orthologs in all three of the tested strains (Data Set S1, worksheet 4), Interestingly, ≥90% of genes upregulated in response to root exudate exposure belonged to the core genome of the three S. meliloti strains (Data Set S1, worksheet 2), suggesting that the large majority of genes required for alfalfa rhizosphere colonization are highly conserved. However, expression patterns were not necessarily conserved, and strain-by-strain and condition-dependent variability of the expression pattern on the conserved gene set was observed (Fig. 4; Fig. S3). Indeed, nested likelihood ratio tests (LRTs) indicated that up to 29% of the conserved genes were influenced by strain-condition interactions, consistent with an important role of GxG interactions in the initiation of rhizobium-legume symbioses (Table 2). Moreover, the same analysis emphasized the role of strain genotype in the response to a common condition (35% of associated DEGs).

**TABLE 2:** Number of expressed genes that showed statistical evidence of each type of expression pattern^*a*^

Parameter	Value for groups of DEGs with significant association								
	Strain	Condition	Strain and condition	Strain × condition^*b*^				None	Total
Model effect									
Strain	*		*	*	*				
Condition		*	*	*		*			
Strain × condition				*	*	*	*		

No. of DEGs (%)	2,028 (25)	87 (1)	1,201 (15)	1,807 A	436 B	34 C	24 D	2,417 (30)	8,034 (100)

a Differential expression related to strain, condition, both strain and condition, or the interaction between strain and condition is reported. Percentages are calculated based on the total number of DEGs. Significance was based on a false discovery rate (FDR)-corrected *P* value of <0.05. * indicates whether the effect of the tested model on the expression of the gene is significant. A gene can be associated with strain (gene differentially expressed only between strains), condition (gene differentially expressed only between different conditions), strain and condition only (gene differentially expressed in relation to strain and condition but not considering the full model strain × condition), or the interaction between strain and condition (strain × condition column). The last situation can be due to a significant association with the three tested models (A), the full model and one of the others (B and C), or the full model only (D).

b The total number of DEGs found for strain and condition was 2,301 (29%).

10.1128/mSystems.00974-20.4 FIG S3 Cluster analysis of the stimulons of the shared set of orthologs. Differentially expressed genes are on the columns. (a) Luteolin; (b) Verbena; (c) Camporegio; (d) Lodi (see File S1 at https://doi.org/10.5061/dryad.jdfn2z38q for interactive heat maps that have each column labeled with the corresponding locus tag). Download FIG S3, PDF file, 0.1 MB. Copyright © 2021 Fagorzi et al. 2021 Fagorzi et al. This content is distributed under the terms of the Creative Commons Attribution 4.0 International license.

### Stimulons differ in the set of elicited functions.

Functional enrichment analyses, based on Kyoto Encyclopedia of Genes and Genomes (KEGG) modules and Clusters of Orthologous Genes (COG) categories, were performed to give a global overview of the functions of the DEGs (Table 3 [see also File S2 at https://doi.org/10.5061/dryad.jdfn2z38q]). Strain- and condition-specific patterns of functional enrichment were observed, consistent with the functional differentiation of the stimulons from each experiment. Nevertheless, a core set of COG categories were commonly over- or underrepresented in all three S. meliloti strains during exposure to the Camporegio or Verbena root exudates. These included enrichment among the upregulated genes of COG categories J and O related to protein expression and modification, suggesting that the root exudates stimulated major remodeling of the proteome. In addition, for upregulated genes, COG category G (carbohydrate transport and metabolism) was underrepresented, while for the downregulated genes, COG category C (energy production and conversion) was overrepresented. This observation suggests that the root exudates stimulated a global change in the cellular energy production pathways versus growth in our standard minimal medium with succinate as the sole carbon source. A comparison with growth under soil-mimicking conditions would be interesting with respect to interpreting the root exudate-induced changes in an ecological context.

**TABLE 3:** Selected COG categories over- or underrepresented among the DEGs^*a*^

COG category	Log_2_ fold change											
	Luteolin			Camporegio			Verbena			Lodi		
	Rm1021	BL225C	AK83	Rm1021	BL225C	AK83	Rm1021	BL225C	AK83	Rm1021	BL225C	AK83
Upregulated genes												
G	—	—	—	−1.14	−1.59	−1.67	−1.30	−1.64	—	—	—	—
J	—	—	—	2.81	0.92	2.50	2.80	1.16	1.81	—	—	—
N	—	—	—	4.19	—	—	4.25	—	—	—	—	—
O	—	—	2.38	1.56	1.44	1.50	1.44	1.91	2.21	—	—	2.96

Downregulated genes												
C	—	—	—	1.88	0.88	1.74	1.89	1.11	1.79	2.71	—	—

a Values represent the log_2_ fold changes in the abundances of genes annotated with the given COG category relative to the amount expected by chance. Dashes indicate that the COG category is not statistically different than chance under the given condition (significance threshold of a *P* value of ≤0.05).

Among the most highly expressed genes in S. meliloti Rm1021 during exposure to the Verbena and Camporegio root exudates were *smc03024* and *smc03028*, encoding components of the flagellar apparatus (*flgF* and *flgC*, respectively); the orthologs of these genes were not induced in BL225C or AK83 (Data Set S1, worksheet 2). The induction of motility is in contrast to the observation that luteolin alone decreases the motility of the S. meliloti Rm1021 strain (50, 51). Presumably, this reflects the presence of additional stimuli in the root exudates. Indeed, amino acids present in root exudates are known to stimulate chemotactic behavior in S. meliloti (52), and signature-tagged mutagenesis showed that motility-related genes are relevant during competition for rhizosphere colonization by S. meliloti Rm1021 (49).

Differences in the transcriptomes of two Bradyrhizobium diazoefficiens strains exposed to root exudates were suggested to be related to differences in their competitive abilities (53). We therefore examined the expression patterns of several genes likely to play a role in competition for rhizosphere colonization and root adhesion. It was previously suggested that the *sin* quorum sensing system is involved in competition in S. meliloti (54); in our data, *sinI* (*smc00168*) was repressed in S. meliloti Rm1021 in the presence of the Camporegio and Verbena root exudates, but no changes in the expression of the orthologous genes in strain AK83 or BL225C were observed. No evidence was found in any of the strains for changes in the expression of galactoglucan or succinoglucan biosynthesis genes such as *wgaA* (*sm_b21319*) and *wgeA* (*sm_b21314*). The Verbena and Camporegio root exudates induced the expression of the rhizobactin transport gene (*sma2337* [*rhtX*]) of Rm1021 and BL225C; this gene is not found in AK83. This may be a consequence of the root exudates chelating the available iron (55), consequently eliciting siderophore production that can inhibit the growth of strains lacking siderophores (56). Plasmid pSINME01 of S. meliloti AK83 exhibits similarity with plasmid pHRC017 of S. meliloti C017, which confers a competitive advantage for nodule occupancy and host range restrictions (57). Considering that a few of the genes on the plasmids pSINME01 and pSINME02 were differentially expressed upon exposure to root exudates, it is possible that the accessory plasmids of strain AK83 also contribute to competition for rhizosphere colonization (57).

Differences in gene expression patterns across conditions may be related, in part, to differences in the presence of flavonoids. In S. meliloti, it is known that root exudates containing flavone molecules activate the transcriptional regulator NodD (43), which triggers the synthesis of Nod factor required for nodule formation. To gain insight into the influence of NodD on the observed stimulons, we compared the S. meliloti Rm1021 data to those of the well-known regulons of NodD1 (requiring plant compounds for its activation) and NodD3 (not requiring plant compounds but relying on indirect activation through SyrM and NodD1 [58]) established previously (51, 59). We found that out of the 26 genes of the NodD1 regulon, 7 and 6 were observed in the DEGs in response to the Verbena and Camporegio root exudates, respectively. Camporegio and Verbena root extracts putatively contained apigenin, while the Lodi root extract lacked apigenin, suggesting a role of apigenin in the differential expression pattern observed. For the 226 genes of the NodD3 regulon, 105, 104, and 4 were found in the DEGs in response to the Verbena, Camporegio, and Lodi root exudates, respectively. The presence of a partial overlap of the known *nod* regulons (∼20% or fewer of the DEGs under each condition) suggests that most of the observed DEGs belong to *nod*-independent regulons. Moreover, some of these genes showed contrasting patterns of expression, suggesting that the root exudates may also contain antagonistic molecules that repress the *nod* regulon, as previously reported (43, 60).

### Mobilization of the symbiotic megaplasmid results in nonadditive changes in stimulons.

To evaluate the impact of interreplicon epistatic interactions on the transcriptional response of S. meliloti to alfalfa root exudates, we used RNA-seq to characterize the response of a previously constructed S. meliloti hybrid strain containing the symbiotic megaplasmid (pSINMEB01) of strain BL225C (48). Cluster analyses clearly demonstrated that the transcriptome (both global and restricted to pSymA-pSINMEB01 orthologs only) of the hybrid strain differed from those of both the BL225C and Rm1021 wild-type strains under all conditions (Fig. 3b; Fig. S4). Of particular interest were the results observed during exposure to the Lodi root exudate. We previously showed that alfalfa cv. Lodi plants inoculated with the hybrid strain were larger than those inoculated with either BL225C or Rm2011 (48). Here, we observed that exposure to the Lodi root exudate results in more differentially expressed genes in the hybrid strain (98 genes) than in either Rm1021 or BL225C (32 and 76 genes, respectively) (Table 1). In particular, a cluster of genes was specifically upregulated in the hybrid strain, and the majority of these genes were located on the symbiotic megaplasmid. This peculiar feature of the Lodi-induced transcriptome in the hybrid strain was also highlighted by the cluster analysis of pSymA-pSINMEB01 orthologs; only in the hybrid strain did the Lodi-induced expression profile cluster with those of the Camporegio and Verbena root exudates (Fig. 3b). The presence of these (possibly nonadditive) transcriptional changes may reflect a loss of *cis*-regulation of these megaplasmid genes by chromosomal regulators (61, 62), providing a potential molecular mechanism underlying the improved symbiotic phenotype of the hybrid compared to both wild-type strains.

10.1128/mSystems.00974-20.5 FIG S4 Clustering of the pSymA hybrid strain with respect to the two parental ones (Rm1021 and BL225C). Heat maps are based on DEGs of orthologous genes. (a) Luteolin; (b) Verbena; (c) Camporegio; (d) Lodi (see File S1 at https://doi.org/10.5061/dryad.jdfn2z38q for interactive heat maps that have each column labeled with the corresponding locus tag). Download FIG S4, PDF file, 0.1 MB. Copyright © 2021 Fagorzi et al. 2021 Fagorzi et al. This content is distributed under the terms of the Creative Commons Attribution 4.0 International license.

## DISCUSSION

Rhizobium-legume interactions are complex multistep phenomena that begin with an exchange of signals between two partners (18, 63). The rhizobia initially detect the plant through the perception of flavonoids in the root exudate of legumes by NodD proteins, which then triggers the production of lipochitooligosaccharide molecules known as Nod factors. Nod factors are then recognized by specific LysM receptor kinase proteins in plant root cells, triggering the symbiosis signaling pathway and initiating the formation of a nodule. However, root exudates contain a mixture of flavonoids, some of them having different agonistic activities on NodD (43). Root exudates also contain many other molecules that can serve as signals or support rhizobium metabolism, such as amino acids and sugars, that may influence the ability of rhizobia to successfully colonize the rhizosphere and be in a position to enter the symbiosis (64, 65). Consequently, interactions between plant and rhizobium genotypes are expected to influence the success of the initial interaction between the two partners.

Previous works have identified a clear role for GxG interactions in the partnership between S. meliloti and Medicago truncatula (66), demonstrating that aerial biomass was influenced by the plant and rhizobium genotypes as well as their interaction. Here, we demonstrated that GxG interactions also have a significant impact on the adherence of S. meliloti strains to alfalfa roots, as a representative phenotype for an early stage of the interaction between these partners. Rhizosphere colonization appears to have a direct impact on nodule colonization (49, 67); while our data do not address if root adhesion is correlated with competition for nodule occupancy in mixed inocula, they suggest that root adhesion is poorly correlated with overall symbiotic efficiency in single-inoculum studies. Previous studies have demonstrated the influence of GxG interactions on the nodule transcriptome of *Medicago*-*Sinorhizobium* symbioses (35, 36). Here, we showed that GxG interactions similarly have an important contribution in determining the transcriptional response of S. meliloti to the detection of Medicago sativa root exudates. Together, these results demonstrate that GxG interactions have a meaningful impact on the outcome of rhizobium-legume symbioses at multiple stages of development.

The exposure of B. diazoefficiens to soybean root exudates resulted in changes in the expression of 450 genes, representing nearly 5.6% of the genome, and the impacts of soybean root exudates differed between the two tested *B. diazoefficiens* strains (53). Similarly, between 0.5% and 20% of S. meliloti genes were differentially expressed following exposure to alfalfa root exudates, depending on the host-symbiont combination. The similarities/differences in the responses of the three S. meliloti strains to treatments did not appear to depend on the phylogenetic relatedness of the strains (68), although this cannot be definitively concluded without analysis of additional strains. Nevertheless, these results emphasize the importance of transcriptional rewiring during strain diversification in bacteria (62). Similarly, studies with eukaryotic organisms indicate that adaptation has an important role in differentiating the gene expression patterns of organisms (69, 70).

The root exudate stimulons only partially overlapped the stimulons of luteolin, a known inducer of NodD in S. meliloti (43), confirming that alfalfa root exudates contain numerous molecular signals aside from flavonoids that may influence the competitiveness of various rhizobium strains. Importantly, the transcriptional patterns induced by alfalfa root exudates differed depending on the cultivar from which they were collected; whether these differences are adaptive requires further investigation. Additionally, although root exudate metabolomic analysis was mainly descriptive, and relatively few peaks could be identified, there was a similar pattern between the differences in the S. meliloti gene expression profiles and the overall chemical similarity of the root exudates as measured by LC-MS; the Camporegio and Verbena root exudates induced similar gene expression changes while also being similar along the second principal component of variance (accounting for 30% of the variance) in the PCA of the root exudate composition. In future work, it would be interesting to define which compounds in the root exudates have the greatest impact on the S. meliloti transcriptome.

In addition to the impact of GxG interactions on rhizobium-legume symbioses, there is the potential for interreplicon interactions within rhizobium genomes to further influence the symbiosis. Indeed, interreplicon epistatic interactions are abundant in the S. meliloti genome (71). To address the contribution of interreplicon interactions to symbiosis, we examined a hybrid strain in which the symbiotic megaplasmid of S. meliloti Rm2011 (a strain nearly identical to Rm1021 [72]) was replaced with the symbiotic megaplasmid of S. meliloti BL225C. Nonadditive effects on the transcriptional profiles associated with all three replicons were observed in the hybrid strain relative to Rm1021 and BL225C, indicating that megaplasmid mobilization induced a global rewiring of gene expression, likely due to transcriptional cross talk among the replicons (62, 73). Similarly, nonadditive effects on the transcriptome of plant hybrids have been extensively explored (74) and demonstrated as one of the bases for heterosis in crops (75). In previous work looking for regulatory modules where the transcription factor and target genes reside on different replicons in S. meliloti Rm1021 (62), we found 17 transcriptional regulators encoded by the chromosome or chromid with predicted target genes on the megaplasmid. Among those transcription factors, systems related to exopolysaccharide production (ExpG), transport (PcaQ), and metabolism (IolR and GlnBK) were present, supporting the hypothesis of a global rewiring of gene expression networks and a wide range of effects of this rewiring. The results with the hybrid led us to hypothesize that the large symbiotic variability observed in natural S. meliloti isolates may partly be related to genome-wide transcriptome changes following large-scale horizontal gene transfer followed by natural selection. Moreover, we speculate that while the megaplasmid is the key element for a general response (i.e., cultivar independent) to species-specific host plant associations, the rhizobium chromosome and chromid fine-tune these responses in a genotype-dependent manner. If true, however, this would limit our ability to predict the competitiveness of rhizobium isolates from their simple genome sequence; instead, a more complex understanding of global regulatory network control would be required.

In conclusion, this study demonstrated that the initial perception of legumes by rhizobia leads to hundreds of changes in the rhizobium transcriptome and that these changes are dependent on the plant genotype, the rhizobium genotype, and genotype-by-genotype interactions. These results complement previous studies demonstrating the role of GxG interactions in determining the transcriptome of both the legume and rhizobium partners in mature N_2_-fixing nodules (35, 36). The majority of genes upregulated in response to alfalfa root exudates were conserved in all three strains, supporting the hypothesis that the S. meliloti lineage was adapted to rhizosphere colonization before gaining the genes required for symbiotic nitrogen fixation (49). Additionally, the transcriptional response to the perception of alfalfa root exudates involved genes from all three of the S. meliloti replicons and seemingly involved nonadditive effects resulting from interreplicon interactions.

## MATERIALS AND METHODS

### Microbiological methods and plant assays.

A list of strains, their host plants of origin, as well as the plant varieties used is reported in Table S4 in the supplemental material. Plant varieties included three contrasting alfalfa genotypes: Medicago falcata (Verbena), M. sativa (Lodi), and *Medicago × varia* (*M. sativa × M. falcata*). Strains included S. meliloti Rm1021, BL225C, AK83, and a hybrid strain containing the chromosome and pSymB of strain Rm2011 and the symbiotic megaplasmid (pSINMEB01) of strain BL225C. S. meliloti Rm2011 is nearly isogenic to Rm1021, both being independent streptomycin-resistant derivatives of the nodule isolate SU47 (76, 77). Details on strains, plant growth, and symbiotic assays are found in Text S1 in the supplemental material. The root adhesion test was performed 5 days following the inoculation of plantlets (Text S1). Differences were evaluated by one-way analysis of variance (ANOVA) Tukey pairwise contrast and using the Scott-Knott procedure as implemented in R (78). All primer pairs used are reported in Table S4.

10.1128/mSystems.00974-20.1 TEXT S1 Details on materials and methods used in this work. Download Text S1, DOCX file, 0.04 MB. Copyright © 2021 Fagorzi et al. 2021 Fagorzi et al. This content is distributed under the terms of the Creative Commons Attribution 4.0 International license.

10.1128/mSystems.00974-20.9 TABLE S4 List of strains (A), alfalfa cultivars (B), and primers (C) used in this study. Download Table S4, PDF file, 0.1 MB. Copyright © 2021 Fagorzi et al. 2021 Fagorzi et al. This content is distributed under the terms of the Creative Commons Attribution 4.0 International license.

### Root exudate production and metabolomic analyses.

Root exudates were produced by growing plants under sterile conditions in water for 14 days, as previously reported (79) and as reported in Text S1. Elemental analysis (CHNS) was performed on crude root exudates (a combined sample for each cultivar) using a carbon, hydrogen, and nitrogen analyzer (CHN-S Flash E1112; Thermo Finnigan, San Jose, CA, USA). Metabolomic analysis was performed by LC-MS, and data from reverse-phase UPLC (RP-UPLC) and UPLC-MS were combined to build the final data matrix. Principal-component analysis (PCA) was performed on the Bray-Curtis dissimilarity obtained from each peak identification (ID) value (Text S1). Statistical differences in single metabolites were assessed by Simper analysis based on the decomposition of the Bray-Curtis dissimilarity obtained from each peak ID value. All statistical analyses were done with the vegan package of R (80). The PubChem database was used for additional peak identification from brute formulas (https://pubchem.ncbi.nlm.nih.gov/).

### RNA isolation and RNA sequencing.

Cultures of S. meliloti, grown overnight in M9-succinate medium at 30°C at 130 rpm, were diluted to an optical density at 600 nm (OD_600_) of 0.05 in 5 ml of M9-succinate medium and incubated until an OD_600_ of 0.4 was reached. Next, either 10 μM luteolin (Sigma-Aldrich) or one of the alfalfa root exudates (normalized by the total organic carbon as measured by the CHNS analysis) was added to each of the cultures, and the mixture was incubated for an additional 4 h at 30°C with shaking at 130 rpm. Biological replicates were performed for each of the three strains across the five conditions. Total RNA was extracted using RNeasy minikits (Qiagen) from 0.5 ml of the culture and subjected to DNase I treatment. Details on the RNA isolation procedure and quality checks are provided in Text S1. Validation of expression differences was done using reverse transcriptase qPCR as described in Text S1. Protocols for rRNA depletion and library construction are described in Text S1. Libraries were sequenced on an Illumina Novaseq 6000 apparatus with an SP flow cell.

### Read mapping, counting, and differential expression analysis.

Trimmed and demultiplexed reads were mapped back to transcripts using Salmon (version 1.1.0) (81) (see Text S1 for details). Quantification files produced by Salmon were then imported into R using the tximport package (version 1.10.1) (82). Differential abundance analysis was performed with the DESeq2 version 1.22.2 package (83) on single strains under different conditions.

### Statistical analysis of differentially expressed genes.

For each S. meliloti strain, genes differentially expressed (log_2_ fold change of ≥1; *P* value of <0.01) under at least one condition relative to the control conditions were identified, and all fold change values for these genes were extracted. To compare expression values of genes conserved between Rm1021, AK83, and BL225C, the pangenome of the three strains was calculated using Roary version 3.13.0 (84) with an identity threshold of 90%, and the genes found in all three strains (the core genes) were recorded. Under each condition, core genes differentially expressed in at least one strain relative to the control conditions were identified, and the fold change values for the gene and its orthologs in the other strains were extracted.

All genes of S. meliloti strains Rm1021, AK83, and BL225C were functionally annotated using stand-alone version 2 of eggNOG-mapper (85, 86) with default settings and the following two modifications: the mode was set to diamond, and query cover was set to 20. Methods for the Kyoto Encyclopedia of Genes and Genomes (KEGG) and Clusters of Orthologous Genes (COG) category annotations are reported in Text S1.

Nested likelihood ratio tests (LRTs) were used to evaluate the statistical significance of strain, condition, and strain-by-condition interaction effects on gene expression. Transcripts were collapsed into orthologous groups based on the output of Roary, as described above. Counts produced by Salmon were collapsed following the group ID provided by Roary, producing a single table with ortholog-level quantification of transcripts. The produced table was then used to perform a nested LRT with DESeq2. Strains and conditions were used together with their interaction to build a model for each group. Terms were then removed one by one to test their impact on the likelihood of the full model (as described in the DESeq2 documentation at http://bioconductor.org/packages/devel/bioc/vignettes/DESeq2/inst/doc/DESeq2.html#likelihood-ratio-test).

### Data availability.

Gene expression data are available at GEO under the accession number GSE151705. Custom scripts developed for this work can be found in the GitHub repository at https://github.com/hyhy8181994/Sinorhizobium-RNAseq-2020.

## References

[1] Rosenberg E, Zilber-Rosenberg I. 2016. Microbes drive evolution of animals and plants: the hologenome concept. mBio 7:e01395-15. doi:10.1128/mBio.01395-15. PMC4817260 27034283

[2] Mus F, Crook MB, Garcia K, Costas AG, Geddes BA, Kouri ED, Paramasivan P, Ryu M-H, Oldroyd GED, Poole PS, Udvardi MK, Voigt CA, Ané J-M, Peters JW. 2016. Symbiotic nitrogen fixation and the challenges to its extension to nonlegumes. Appl Environ Microbiol 82:3698–3710. doi:10.1128/AEM.01055-16. 27084023 PMC4907175

[3] Lee YK, Mazmanian SK. 2010. Has the microbiota played a critical role in the evolution of the adaptive immune system? Science 330:1768–1773. doi:10.1126/science.1195568. 21205662 PMC3159383

[4] Jones BW, Nishiguchi MK. 2004. Counterillumination in the Hawaiian bobtail squid, Euprymna scolopes Berry (Mollusca: Cephalopoda). Mar Biol 144:1151–1155. doi:10.1007/s00227-003-1285-3.

[5] Nyholm SV, McFall-Ngai M. 2004. The winnowing: establishing the squid-vibrio symbiosis. Nat Rev Microbiol 2:632–642. doi:10.1038/nrmicro957. 15263898

[6] Kiers ET, Rousseau RA, West SA, Denison RF. 2003. Host sanctions and the legume-rhizobium mutualism. Nature 425:78–81. doi:10.1038/nature01931. 12955144

[7] Mwafulirwa L, Baggs EM, Russell J, George T, Morley N, Sim A, de la Fuente Cantó C, Paterson E. 2016. Barley genotype influences stabilization of rhizodeposition-derived C and soil organic matter mineralization. Soil Biol Biochem 95:60–69. doi:10.1016/j.soilbio.2015.12.011.

[8] Escudero-Martinez C, Bulgarelli D. 2019. Tracing the evolutionary routes of plant-microbiota interactions. Curr Opin Microbiol 49:34–40. doi:10.1016/j.mib.2019.09.013. 31698159

[9] Pasolli E, Asnicar F, Manara S, Zolfo M, Karcher N, Armanini F, Beghini F, Manghi P, Tett A, Ghensi P, Collado MC, Rice BL, DuLong C, Morgan XC, Golden CD, Quince C, Huttenhower C, Segata N. 2019. Extensive unexplored human microbiome diversity revealed by over 150,000 genomes from metagenomes spanning age, geography, and lifestyle. Cell 176:649–662.e20. doi:10.1016/j.cell.2019.01.001. 30661755 PMC6349461

[10] Singh BK, Liu H, Trivedi P. 2020. Eco-holobiont: a new concept to identify drivers of host-associated microorganisms. Environ Microbiol 22:564–567. doi:10.1111/1462-2920.14900. 31849163

[11] de Souza RSC, Armanhi JSL, Damasceno NDB, Imperial J, Arruda P. 2019. Genome sequences of a plant beneficial synthetic bacterial community reveal genetic features for successful plant colonization. Front Microbiol 10:1779. doi:10.3389/fmicb.2019.01779. 31456759 PMC6701196

[12] Levy A, Gonzalez IS, Mittelviefhaus M, Clingenpeel S, Paredes SH, Miao J, Wang K, Devescovi G, Stillman K, Monteiro F, Alvarez BR, Lundberg DS, Lu T-Y, Lebeis S, Jin Z, McDonald M, Klein AP, Feltcher ME, Rio TG, Grant SR, Doty SL, Ley RE, Zhao B, Venturi V, Pelletier DA, Vorholt JA, Tringe SG, Woyke T, Dangl JL. 2018. Genomic features of bacterial adaptation to plants. Nat Genet 50:138–150. doi:10.1038/s41588-017-0012-9. PMC5957079 29255260

[13] Harrison E, Brockhurst MA. 2012. Plasmid-mediated horizontal gene transfer is a coevolutionary process. Trends Microbiol 20:262–267. doi:10.1016/j.tim.2012.04.003. 22564249

[14] Batstone RT, O’Brien AM, Harrison TL, Frederickson ME. 2020. Experimental evolution makes microbes more cooperative with their local host genotype. Science 370:476–478. doi:10.1126/science.abb7222. 33093112

[15] Pini F, Galardini M, Bazzicalupo M, Mengoni A. 2011. Plant-bacteria association and symbiosis: are there common genomic traits in Alphaproteobacteria? Genes (Basel) 2:1017–1032. doi:10.3390/genes2041017. 24710303 PMC3927591

[16] Guttman D, McHardy AC, Schulze-Lefert P. 2014. Microbial genome-enabled insights into plant-microorganism interactions. Nat Rev Genet 15:797–813. doi:10.1038/nrg3748. 25266034

[17] Poole P, Ramachandran V, Terpolilli J. 2018. Rhizobia: from saprophytes to endosymbionts. Nat Rev Microbiol 16:291–303. doi:10.1038/nrmicro.2017.171. 29379215

[18] Oldroyd GED. 2013. Speak, friend, and enter: signalling systems that promote beneficial symbiotic associations in plants. Nat Rev Microbiol 11:252–263. doi:10.1038/nrmicro2990. 23493145

[19] Gage DJ. 2004. Infection and invasion of roots by symbiotic, nitrogen-fixing rhizobia during nodulation of temperate legumes. Microbiol Mol Biol Rev 68:280–300. doi:10.1128/MMBR.68.2.280-300.2004. 15187185 PMC419923

[20] Udvardi M, Poole PS. 2013. Transport and metabolism in legume-rhizobia symbioses. Annu Rev Plant Biol 64:781–805. doi:10.1146/annurev-arplant-050312-120235. 23451778

[21] Kereszt A, Mergaert P, Kondorosi E. 2011. Bacteroid development in legume nodules: evolution of mutual benefit or of sacrificial victims? Mol Plant Microbe Interact 24:1300–1309. doi:10.1094/MPMI-06-11-0152. 21995798

[22] Benezech C, Doudement M, Gourion B. 2020. Legumes tolerance to rhizobia is not always observed and not always deserved. Cell Microbiol 22:e13124. doi:10.1111/cmi.13124. 31610071

[23] Gibson KE, Kobayashi H, Walker GC. 2008. Molecular determinants of a symbiotic chronic infection. Annu Rev Genet 42:413–441. doi:10.1146/annurev.genet.42.110807.091427. 18983260 PMC2770587

[24] Burghardt LT. 2020. Evolving together, evolving apart: measuring the fitness of rhizobial bacteria in and out of symbiosis with leguminous plants. New Phytol 228:28–34. doi:10.1111/nph.16045. 31276218

[25] Paffetti D, Scotti C, Gnocchi S, Fancelli S, Bazzicalupo M. 1996. Genetic diversity of an Italian Rhizobium meliloti population from different Medicago sativa varieties. Appl Environ Microbiol 62:2279–2285. doi:10.1128/AEM.62.7.2279-2285.1996. 8779566 PMC168009

[26] Carelli M, Gnocchi S, Fancelli S, Mengoni A, Paffetti D, Scotti C, Bazzicalupo M. 2000. Genetic diversity and dynamics of Sinorhizobium meliloti populations nodulating different alfalfa cultivars in Italian soils. Appl Environ Microbiol 66:4785–4789. doi:10.1128/aem.66.11.4785-4789.2000. 11055924 PMC92380

[27] Rangin C, Brunel B, Cleyet-Marel J-C, Perrineau M-M, Béna G. 2008. Effects of Medicago truncatula genetic diversity, rhizobial competition, and strain effectiveness on the diversity of a natural Sinorhizobium species community. Appl Environ Microbiol 74:5653–5661. doi:10.1128/AEM.01107-08. 18658290 PMC2547051

[28] Heath KD, Tiffin P. 2007. Context dependence in the coevolution of plant and rhizobial mutualists. Proc Biol Sci 274:1905–1912. doi:10.1098/rspb.2007.0495. 17535796 PMC2270936

[29] Heath KD, Tiffin P. 2009. Stabilizing mechanisms in a legume-rhizobium mutualism. Evolution 63:652–662. doi:10.1111/j.1558-5646.2008.00582.x. 19087187

[30] Heath KD. 2010. Intergenomic epistasis and coevolutionary constraint in plants and rhizobia. Evolution 64:1446–1458. doi:10.1111/j.1558-5646.2009.00913.x. 20002161

[31] Ehinger M, Mohr TJ, Starcevich JB, Sachs JL, Porter SS, Simms EL. 2014. Specialization-generalization trade-off in a Bradyrhizobium symbiosis with wild legume hosts. BMC Ecol 14:8. doi:10.1186/1472-6785-14-8. 24641813 PMC4021497

[32] Burghardt LT, Epstein B, Guhlin J, Nelson MS, Taylor MR, Young ND, Sadowsky MJ, Tiffin P. 2018. Select and resequence reveals relative fitness of bacteria in symbiotic and free-living environments. Proc Natl Acad Sci U S A 115:2425–2430. doi:10.1073/pnas.1714246115. 29453274 PMC5877963

[33] Burghardt LT, Trujillo DI, Epstein B, Tiffin P, Young ND. 2020. A select and resequence approach reveals strain-specific effects of Medicago nodule-specific PLAT-domain genes. Plant Physiol 182:463–471. doi:10.1104/pp.19.00831. 31653715 PMC6945875

[34] Roux B, Rodde N, Jardinaud MF, Timmers T, Sauviac L, Cottret L, Carrère S, Sallet E, Courcelle E, Moreau S, Debellé F, Capela D, De Carvalho-Niebel F, Gouzy J, Bruand C, Gamas P. 2014. An integrated analysis of plant and bacterial gene expression in symbiotic root nodules using laser-capture microdissection coupled to RNA sequencing. Plant J 77:817–837. doi:10.1111/tpj.12442. 24483147

[35] Heath KD, Burke PV, Stinchcombe JR. 2012. Coevolutionary genetic variation in the legume-rhizobium transcriptome. Mol Ecol 21:4735–4747. doi:10.1111/j.1365-294X.2012.05629.x. 22672103

[36] Burghardt LT, Guhlin J, Chun CL, Liu J, Sadowsky MJ, Stupar RM, Young ND, Tiffin P. 2017. Transcriptomic basis of genome by genome variation in a legume-rhizobia mutualism. Mol Ecol 26:6122–6135. doi:10.1111/mec.14285. 28792680

[37] Triplett EW, Sadowsky MJ. 1992. Genetics of competition for nodulation of legumes. Annu Rev Microbiol 46:399–422. doi:10.1146/annurev.mi.46.100192.002151. 1444262

[38] Checcucci A, DiCenzo GC, Bazzicalupo M, Mengoni A. 2017. Trade, diplomacy, and warfare: the quest for elite rhizobia inoculant strains. Front Microbiol 8:2207. doi:10.3389/fmicb.2017.02207. 29170661 PMC5684177

[39] Sprent JI, Ardley J, James EK. 2017. Biogeography of nodulated legumes and their nitrogen-fixing symbionts. New Phytol 215:40–56. doi:10.1111/nph.14474. 28211601

[40] Frame J, Charlton JFL, Laidlaw AS. 1998. Temperate forage legumes. CAB International, Wallingford, United Kingdom.

[41] Galibert F, Finan TM, Long SR, Puhler A, Abola P, Ampe F, Barloy-Hubler F, Barnett MJ, Becker A, Boistard P, Bothe G, Boutry M, Bowser L, Buhrmester J, Cadieu E, Capela D, Chain P, Cowie A, Davis RW, Dreano S, Federspiel NA, Fisher RF, Gloux S, Godrie T, Goffeau A, Golding B, Gouzy J, Gurjal M, Hernandez-Lucas I, Hong A, Huizar L, Hyman RW, Jones T, Kahn D, Kahn ML, Kalman S, Keating DH, Kiss E, Komp C, Lelaure V, Masuy D, Palm C, Peck MC, Pohl TM, Portetelle D, Purnelle B, Ramsperger U, Surzycki R, Thebault P, Vandenbol M, Vorholter FJ, . 2001. The composite genome of the legume symbiont Sinorhizobium meliloti. Science 293:668–672. doi:10.1126/science.1060966. 11474104

[42] Harrison PW, Lower RPJ, Kim NKD, Young JPW. 2010. Introducing the bacterial “chromid”: not a chromosome, not a plasmid. Trends Microbiol 18:141–148. doi:10.1016/j.tim.2009.12.010. 20080407

[43] Peck MC, Fisher RF, Long SR. 2006. Diverse flavonoids stimulate NodD1 binding to nod gene promoters in Sinorhizobium meliloti. J Bacteriol 188:5417–5427. doi:10.1128/JB.00376-06. 16855231 PMC1540014

[44] Galardini M, Mengoni A, Brilli M, Pini F, Fioravanti A, Lucas S, Lapidus A, Cheng J-F, Goodwin L, Pitluck S, Land M, Hauser L, Woyke T, Mikhailova N, Ivanova N, Daligault H, Bruce D, Detter C, Tapia R, Han C, Teshima H, Mocali S, Bazzicalupo M, Biondi EG. 2011. Exploring the symbiotic pangenome of the nitrogen-fixing bacterium Sinorhizobium meliloti. BMC Genomics 12:235. doi:10.1186/1471-2164-12-235. 21569405 PMC3164228

[45] Biondi EG, Tatti E, Comparini D, Giuntini E, Mocali S, Giovannetti L, Bazzicalupo M, Mengoni A, Viti C. 2009. Metabolic capacity of Sinorhizobium (Ensifer) meliloti strains as determined by phenotype microarray analysis. Appl Environ Microbiol 75:5396–5404. doi:10.1128/AEM.00196-09. 19561177 PMC2725449

[46] Checcucci A, Azzarello E, Bazzicalupo M, Galardini M, Lagomarsino A, Mancuso S, Marti L, Marzano MC, Mocali S, Squartini A, Zanardo M, Mengoni A. 2016. Mixed nodule infection in Sinorhizobium meliloti-Medicago sativa symbiosis suggest the presence of cheating behavior. Front Plant Sci 7:835. doi:10.3389/fpls.2016.00835. 27379128 PMC4904023

[47] Caetano-Anollés G, Crist-Estes DK, Bauer WD. 1988. Chemotaxis of Rhizobium meliloti to the plant flavone luteolin requires functional nodulation genes. J Bacteriol 170:3164–3169. doi:10.1128/jb.170.7.3164-3169.1988. 3384804 PMC211264

[48] Checcucci A, DiCenzo GC, Ghini V, Bazzicalupo M, Becker A, Decorosi F, Döhlemann J, Fagorzi C, Finan TM, Fondi M, Luchinat C, Turano P, Vignolini T, Viti C, Mengoni A. 2018. Creation and characterization of a genomically hybrid strain in the nitrogen-fixing symbiotic bacterium Sinorhizobium meliloti. ACS Synth Biol 7:2365–2378. doi:10.1021/acssynbio.8b00158. 30223644

[49] Salas ME, Lozano MJ, López JL, Draghi WO, Serrania J, Torres Tejerizo GA, Albicoro FJ, Nilsson JF, Pistorio M, Del Papa MF, Parisi G, Becker A, Lagares A. 2017. Specificity traits consistent with legume-rhizobia coevolution displayed by Ensifer meliloti rhizosphere colonization. Environ Microbiol 19:3423–3438. doi:10.1111/1462-2920.13820. 28618121

[50] Spini G, Decorosi F, Cerboneschi M, Tegli S, Mengoni A, Viti C, Giovannetti L. 2016. Effect of the plant flavonoid luteolin on Ensifer meliloti 3001 phenotypic responses. Plant Soil 399:159–178. doi:10.1007/s11104-015-2659-2.

[51] Barnett MJ, Long SR. 2015. The Sinorhizobium meliloti SyrM regulon: effects on global gene expression are mediated by syrA and nodD3. J Bacteriol 197:1792–1806. doi:10.1128/JB.02626-14. 25777671 PMC4402393

[52] Webb BA, Helm RF, Scharf BE. 2016. Contribution of individual chemoreceptors to Sinorhizobium meliloti chemotaxis towards amino acids of host and nonhost seed exudates. Mol Plant Microbe Interact 29:231–239. doi:10.1094/MPMI-12-15-0264-R. 26713349

[53] Liu Y, Jiang X, Guan D, Zhou W, Ma M, Zhao B, Cao F, Li L, Li J. 2017. Transcriptional analysis of genes involved in competitive nodulation in Bradyrhizobium diazoefficiens at the presence of soybean root exudates. Sci Rep 7:10946. doi:10.1038/s41598-017-11372-0. 28887528 PMC5591287

[54] McIntosh M, Krol E, Becker A. 2008. Competitive and cooperative effects in quorum-sensing-regulated galactoglucan biosynthesis in Sinorhizobium meliloti. J Bacteriol 190:5308–5317. doi:10.1128/JB.00063-08. 18515420 PMC2493264

[55] Parker DR, Reichman SM, Crowley DE. 2005. Metal chelation in the rhizosphere, p 57–93. *In* Zobel RW, Wright SF (ed), Roots and soil management: interactions between roots and the soil, vol 48. American Society of Agronomy, Inc, Madison, WI.

[56] diCenzo GC, MacLean AM, Milunovic B, Golding GB, Finan TM. 2014. Examination of prokaryotic multipartite genome evolution through experimental genome reduction. PLoS Genet 10:e1004742. doi:10.1371/journal.pgen.1004742. 25340565 PMC4207669

[57] Crook MB, Lindsay DP, Biggs MB, Bentley JS, Price JC, Clement SC, Clement MJ, Long SR, Griffitts JS. 2012. Rhizobial plasmids that cause impaired symbiotic nitrogen fixation and enhanced host invasion. Mol Plant Microbe Interact 25:1026–1033. doi:10.1094/MPMI-02-12-0052-R. 22746823 PMC4406224

[58] Maillet F, Debellé F, Dénarié J. 1990. Role of the nodD and syrM genes in the activation of the regulatory gene nodD3, and of the common and host‐specific nod genes of Rhizobium meliloti. Mol Microbiol 4:1975–1984. doi:10.1111/j.1365-2958.1990.tb02047.x. 2127953

[59] Capela D, Carrere S, Batut J. 2005. Transcriptome-based identification of the Sinorhizobium meliloti NodD1 regulon. Appl Environ Microbiol 71:4910–4913. doi:10.1128/AEM.71.8.4910-4913.2005. 16085895 PMC1183327

[60] Zuanazzi JAS, Clergeot PH, Quirion J-C, Husson H-P, Kondorosi A, Ratet P. 1998. Production of Sinorhizobium meliloti nod gene activator and repressor flavonoids from Medicago sativa roots. Mol Plant Microbe Interact 11:784–794. doi:10.1094/MPMI.1998.11.8.784.

[61] Ramos JL, Marqués S, Timmis KN. 1997. Transcriptional control of the Pseudomonas TOL plasmid catabolic operons is achieved through an interplay of host factors and plasmid-encoded regulators. Annu Rev Microbiol 51:341–373. doi:10.1146/annurev.micro.51.1.341. 9343354

[62] Galardini M, Brilli M, Spini G, Rossi M, Roncaglia B, Bani A, Chiancianesi M, Moretto M, Engelen K, Bacci G, Pini F, Biondi EG, Bazzicalupo M, Mengoni A. 2015. Evolution of intra-specific regulatory networks in a multipartite bacterial genome. PLoS Comput Biol 11:e1004478. doi:10.1371/journal.pcbi.1004478. 26340565 PMC4560400

[63] Oldroyd GED, Murray JD, Poole PS, Downie JA. 2011. The rules of engagement in the legume-rhizobial symbiosis. Annu Rev Genet 45:119–144. doi:10.1146/annurev-genet-110410-132549. 21838550

[64] Barbour WM, Hattermann DR, Stacey G. 1991. Chemotaxis of Bradyrhizobium japonicum to soybean exudates. Appl Environ Microbiol 57:2635–2639. doi:10.1128/AEM.57.9.2635-2639.1991. 1768137 PMC183632

[65] Webb BA, Compton KK, Martin JS, Taylor D, Sobrado P, Scharf BE. 2017. Sinorhizobium meliloti chemotaxis to multiple amino acids is mediated by the chemoreceptor McpU. Mol Plant Microbe Interact 30:770–777. doi:10.1094/MPMI-04-17-0096-R. 28745538

[66] Mhadhbi H, Jebara M, Limam F, Huguet T, Aouani ME. 2005. Interaction between Medicago truncatula lines and Sinorhizobium meliloti strains for symbiotic efficiency and nodule antioxidant activities. Physiol Plant 124:4–11. doi:10.1111/j.1399-3054.2005.00489.x.

[67] Entcheva P, Phillips DA, Streit WR. 2002. Functional analysis of Sinorhizobium meliloti genes involved in biotin synthesis and transport. Appl Environ Microbiol 68:2843–2848. doi:10.1128/aem.68.6.2843-2848.2002. 12039741 PMC123963

[68] Galardini M, Pini F, Bazzicalupo M, Biondi EG, Mengoni A. 2013. Replicon-dependent bacterial genome evolution: the case of Sinorhizobium meliloti. Genome Biol Evol 5:542–558. doi:10.1093/gbe/evt027. 23431003 PMC3622305

[69] López-Maury L, Marguerat S, Bähler J. 2008. Tuning gene expression to changing environments: from rapid responses to evolutionary adaptation. Nat Rev Genet 9:583–593. doi:10.1038/nrg2398. 18591982

[70] Whitehead A, Crawford DL. 2006. Variation within and among species in gene expression: raw material for evolution. Mol Ecol 15:1197–1211. doi:10.1111/j.1365-294X.2006.02868.x. 16626448

[71] diCenzo GC, Benedict AB, Fondi M, Walker GC, Finan TM, Mengoni A, Griffitts JS. 2018. Robustness encoded across essential and accessory replicons of the ecologically versatile bacterium Sinorhizobium meliloti. PLoS Genet 14:e1007357. doi:10.1371/journal.pgen.1007357. 29672509 PMC5929573

[72] Meade HM, Signer ER. 1977. Genetic mapping of Rhizobium meliloti. Proc Natl Acad Sci U S A 74:2076–2078. doi:10.1073/pnas.74.5.2076. 266730 PMC431077

[73] diCenzo GC, Wellappili D, Golding GB, Finan TM. 2018. Inter-replicon gene flow contributes to transcriptional integration in the *Sinorhizobium meliloti* multipartite genome. G3 (Bethesda) 8:1711–1720. doi:10.1534/g3.117.300405. 29563186 PMC5940162

[74] Bell GDM, Kane NC, Rieseberg LH, Adams KL. 2013. RNA-seq analysis of allele-specific expression, hybrid effects, and regulatory divergence in hybrids compared with their parents from natural populations. Genome Biol Evol 5:1309–1323. doi:10.1093/gbe/evt072. 23677938 PMC3730339

[75] Hochholdinger F, Hoecker N. 2007. Towards the molecular basis of heterosis. Trends Plant Sci 12:427–432. doi:10.1016/j.tplants.2007.08.005. 17720610

[76] Sallet E, Roux B, Sauviac L, Jardinaud MF, Carrère S, Faraut T, de Carvalho-Niebel F, Gouzy J, Gamas P, Capela D, Bruand C, Schiex T. 2013. Next-generation annotation of prokaryotic genomes with EuGene-P: application to Sinorhizobium meliloti 2011. DNA Res 20:339–353. doi:10.1093/dnares/dst014. 23599422 PMC3738161

[77] Krol E, Becker A. 2004. Global transcriptional analysis of the phosphate starvation response in Sinorhizobium meliloti strains 1021 and 2011. Mol Genet Genomics 272:1–17. doi:10.1007/s00438-004-1030-8. 15221452

[78] R Development Core Team. 2012. R: a language and environment for statistical computing. R Foundation for Statistical Computing, Vienna, Austria.

[79] Checcucci A, Azzarello E, Bazzicalupo M, De Carlo A, Emiliani G, Mancuso S, Spini G, Viti C, Mengoni A. 2017. Role and regulation of ACC deaminase gene in Sinorhizobium meliloti: is it a symbiotic, rhizospheric or endophytic gene? Front Genet 8:6. doi:10.3389/fgene.2017.00006. 28194158 PMC5276845

[80] Oksanen J, Blanchet F, Kindt R, Legendre P, Minchin P, O’Hara R, Simpson G, Solymos P, Stevens M, Wagner H. 2013. vegan: community ecology package. R package version 2.0-10.

[81] Patro R, Duggal G, Love MI, Irizarry RA, Kingsford C. 2017. Salmon provides fast and bias-aware quantification of transcript expression. Nat Methods 14:417–419. doi:10.1038/nmeth.4197. 28263959 PMC5600148

[82] Soneson C, Love MI, Robinson MD. 2015. Differential analyses for RNA-seq: transcript-level estimates improve gene-level inferences. F1000Res 4:1521. doi:10.12688/f1000research.7563.1. 26925227 PMC4712774

[83] Love MI, Huber W, Anders S. 2014. Moderated estimation of fold change and dispersion for RNA-seq data with DESeq2. Genome Biol 15:550. doi:10.1186/s13059-014-0550-8. 25516281 PMC4302049

[84] Page AJ, Cummins CA, Hunt M, Wong VK, Reuter S, Holden MTG, Fookes M, Falush D, Keane JA, Parkhill J. 2015. Roary: rapid large-scale prokaryote pan genome analysis. Bioinformatics 31:3691–3693. doi:10.1093/bioinformatics/btv421. 26198102 PMC4817141

[85] Huerta-Cepas J, Szklarczyk D, Heller D, Hernández-Plaza A, Forslund SK, Cook H, Mende DR, Letunic I, Rattei T, Jensen LJ, von Mering C, Bork P. 2019. eggNOG 5.0: a hierarchical, functionally and phylogenetically annotated orthology resource based on 5090 organisms and 2502 viruses. Nucleic Acids Res 47:D309–D314. doi:10.1093/nar/gky1085. 30418610 PMC6324079

[86] Huerta-Cepas J, Forslund K, Coelho LP, Szklarczyk D, Jensen LJ, Von Mering C, Bork P. 2017. Fast genome-wide functional annotation through orthology assignment by eggNOG-mapper. Mol Biol Evol 34:2115–2122. doi:10.1093/molbev/msx148. 28460117 PMC5850834

