# Supplementary figures and images for: Nonadditive Transcriptomic Signatures of Genotype-by-Genotype Interactions during the Initiation of Plant-Rhizobium Symbiosis
Source: mSystems. 2021 Jan 12;6(1):e00974-20. doi: 10.1128/mSystems.00974-20 (PMC7901481; doi:10.1128/mSystems.00974-20)

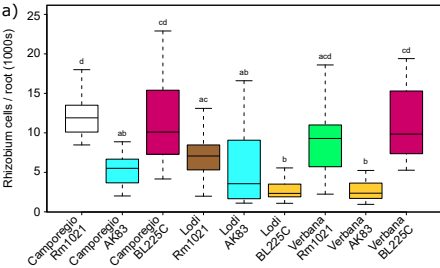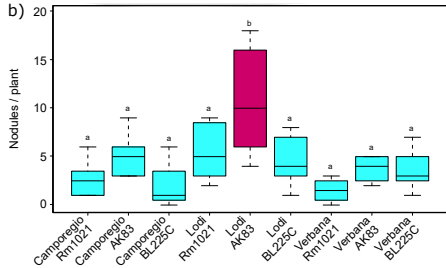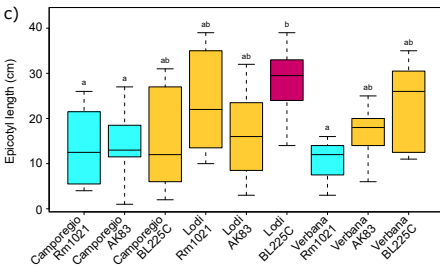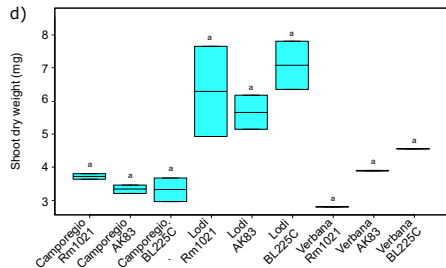

Supplement: FIG S1 [file mSystems.00974-20-sf001.pdf]

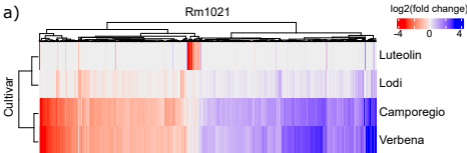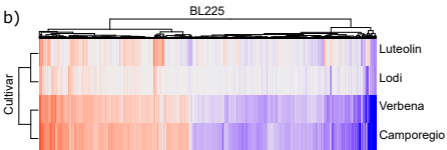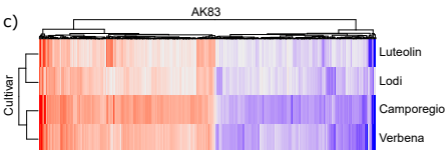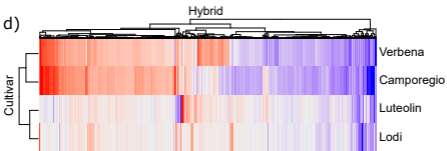

Supplement: FIG S2 [file mSystems.00974-20-sf002.pdf]

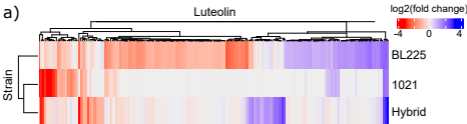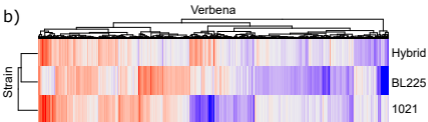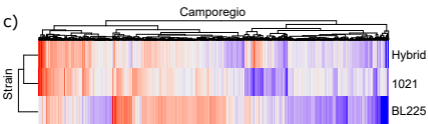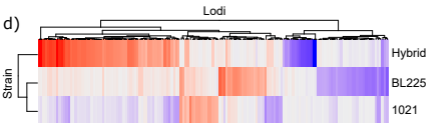

Supplement: FIG S3 [file mSystems.00974-20-sf003.pdf]

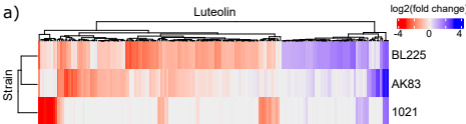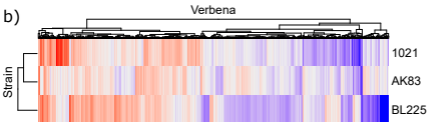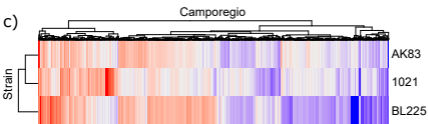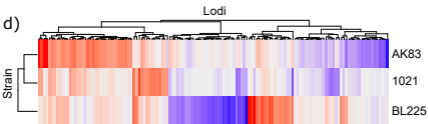

Supplement: FIG S4 [file mSystems.00974-20-sf004.pdf]
