# Supplementary material for: Nonadditive Transcriptomic Signatures of Genotype-by-Genotype Interactions during the Initiation of Plant-Rhizobium Symbiosis
Source: mSystems. 2021 Jan 12;6(1):e00974-20. doi: 10.1128/mSystems.00974-20 (PMC7901481; doi:10.1128/mSystems.00974-20)
Supplement: TABLE S1 [file mSystems.00974-20-st001.pdf]

Table S1.

Camporegio vs. Lodi

Peak ID	Av. dissim	Contrib. %	Hypothetical compound
PP_11707	21.08	29.16	N-Acetyl-L-leucine
PP_16427	8.518	11.79	DL-Tryptophan
PP_01693	5.773	7.988	Cytosine

Camporegio vs. Verbena

Peak ID	Av. dissim	Contrib. %	Hypothetical compound
PP_11707	17.08	24.3	N-Acetyl-L-leucine
PP_16427	6.941	9.876	DL-Tryptophan
PP_01693	4.187	5.957	Cytosine

Lodi vs. Verbena

Peak ID	Av. dissim	Contrib. %	Hypothetical compound
PP_13281	10.57	21.59	3,5-Dihydroxyphenylglycine
PP_14042	2.207	4.508	Val-Ala
PP_11707	1.995	4.075	N-Acetyl-L-leucine
