# Supplementary material for: Nonadditive Transcriptomic Signatures of Genotype-by-Genotype Interactions during the Initiation of Plant-Rhizobium Symbiosis
Source: mSystems. 2021 Jan 12;6(1):e00974-20. doi: 10.1128/mSystems.00974-20 (PMC7901481; doi:10.1128/mSystems.00974-20)
Supplement: TABLE S2 [file mSystems.00974-20-st002.pdf]

Table S2.

	N%	C%	H%
**Blank**	n.d.	0.017	0.49
**Lodi**	0.017	0.246	56.61
**Verbena**	n.d.	0.060	55.47
**Camporegio**	n.d.	0.056	30.05
