# Supplementary material for: Nonadditive Transcriptomic Signatures of Genotype-by-Genotype Interactions during the Initiation of Plant-Rhizobium Symbiosis
Source: mSystems. 2021 Jan 12;6(1):e00974-20. doi: 10.1128/mSystems.00974-20 (PMC7901481; doi:10.1128/mSystems.00974-20)
Supplement: TEXT S1 [file mSystems.00974-20-s0001.docx]

**Supplemental Information file**

**Dissecting transcriptomic signatures of genotype x genotype interactions during the initiation of plant-rhizobium symbiosis**

Camilla Fagorzi^a^, Giovanni Bacci^a,#^, Rui Huang^b^, Lisa Cangioli^a^, Alice Checcucci^a,*^, Margherita Fini^a^, Elena Perrin^a^, Chiara Natali^a^, George Colin diCenzo^b^, Alessio Mengoni^a,#^

^a^ Dipartimento di Biologia, University of Florence, Florence, Italy

^b^ Department of Biology, Queen’s University, Kingston, Ontario, Canada

# Address correspondence to Alessio Mengoni:[alessio.mengoni@unifi.it](mailto:alessio.mengoni@unifi.it) and to Giovanni Bacci, [giovanni.bacci@unifi.it](mailto:giovanni.bacci@unifi.it) (computational part)

^*^ present address, Department of Agricultural and Food Science, University of Bologna, Bologna, Italy

**Strains and microbiological methods**

*S. meliloti* Rm1021 is a spontaneous streptomycin-resistant derivative of the isolate SU47 recovered form *M. sativa* root nodules (1). *S. meliloti* BL225C was isolated by plant trapping with the *M. sativa* variety “Lodi” in Lodi, Italy in 1996 (2). *S. meliloti* strain AK83 was isolated from nodules of *M. falcata* grown in soil samples from the North Aral Sea region in Kazakhstan in 2001 (3). *S. meliloti* BM806 (later termed as “hybrid strain”) is a Rm2011 (a near identical strain to Rm1021, as both are independent streptomycin resistant derivatives of the nodule isolate *S. meliloti* SU47 (4)) derivative in which the pSymA megaplasmid was replaced with the homologous megaplasmid (pSINMEB01) from strain BL225C (5). Strains were grown at 30°C in TY with 0.2 g/l CaCl_2_, or in M9 supplemented with 0.2% succinate as the carbon source. For Rm1021, streptomycin (200 µg/mL) was added to the culture medium during routine growth.

**Plant varieties, root adhesion tests, and symbiotic assays**

Fall dormancy (FD) is an important trait having large impacts on the productivity and persistence of alfalfa (6). Cultivars Camporegio and Verbena are included in the subgroup of fall dormant type (FDT; FD 1–4), while cultivar Lodi is a semi-dormant type (SDT; FD 5–7). Symbiotic assays were performed, as previously reported (5), on 12 plants per strain – cultivar combination. Briefly, plants were grown in pots containing a 1:1 mixture of sterile vermiculite and perlite, supplemented with 200 mL of Fahraeus N-free liquid and inoculated with ∼4 ×10^4^ rhizobial cells/cm^3^ and grown in a growth chamber at 26 °C with a 16 h photoperiod (100 microeinstein/m^2^/s) for 5 weeks. Nodule counts were performed after the 5 weeks, then the shoots dried at 50 °C for 7 days. The root adhesion test was performed five days following the inoculation of plantlets. Using sterile tweezers, plantlets were carefully removed from the substrate and divided into epicotyl and hypocotyl (i.e. root) portions. After measuring their length, roots of similar length (~ 3 cm) were washed to remove loosely adherent cells by vortexing for ten seconds in 500 μl of 0.9% NaCl. Then, roots were transferred to 500 µl of fresh 0.9% NaCl, and vortexed for 30 seconds to collect bacterial cells that were strongly adhered to the root surface. Roots were removed from the tube and the quantity of bacterial cells detached from the roots and recovered in the NaCl solution was evaluated using Real Time PCR (qPCR) by a standard curve method on the *nodB* gene in a QuantStudio^TM^ 7 flex (Applied Biosystems), as previously described (7, 8). Differences were evaluated by one-way ANOVA Tukey pairwise contrast and using the Scott-Knott procedure as implemented in R (9).

**Root exudate production and metabolomic analyses**

Root exudates were produced growing plantlets in water as previously reported (10) in two independent experiments, giving two biological replicas for each plant cultivar. A blank sample was prepared with the same setup of the plant experiment, but without adding the plantlet. Elemental analysis (CHNS) was performed on crude root exudates (a combined sample for each cultivar) using a carbon hydrogen and nitrogen analyzer (CHN-S Flash E1112, Thermofinnigan, San José, California, United States). For LC-MS, the extraction of the seven samples (two biological replicates per cultivar, plus the blank) was performed by metaSysX GmbH ([www.metasysx.com](http://www.metasysx.com)) with a modified protocol from (11). The samples were measured with a Waters ACQUITY Reversed Phase Ultra Performance Liquid Chromatography (RP-UPLC) coupled to a Thermo-Fisher Q-Exactive mass spectrometer that consists of an ElectroSpray Ionization source (ESI) and an Orbitrap mass analyzer (UPLC-MS). A C18 column was used for the chromatographic separation of the hydrophilic compounds. The mass spectra were acquired in full scan MS positive and negative modes (Mass Range [100−1500]). Extraction of the LC-MS data was accomplished with the software REFINER MS® 10.5 (GeneData, [genedata.com](http://www.genedata.com)). After extraction of the peak list from the chromatograms, data were processed, aligned, and filtered using in-house software. Only those features (peak IDs) that were present in at least two out of the seven samples were kept. At this stage, an average retention time (RT) and average m/z values were given to each feature. The alignment was performed for each platform independently (polar phase positive mode, polar phase negative mode). The annotation of the content of the sample was accomplished by matching the extracted data from the chromatograms with metaSysX’s library of reference compounds. Data from both platforms (RP-UPLC and UPLC-MS) were combined to build the final data matrix. Attribution of brute formulas to known compound was done using the PubChem database ([pubchem.ncbi.nlm.nih.gov/](https://pubchem.ncbi.nlm.nih.gov/)). Principal Component Analysis (PCA) was performed on the Bray-Curtis dissimilarity obtained from each peak ID value. Statistical differences in single metabolites were assessed by Simper analysis based on the decomposition of the Bray-Curtis dissimilarity obtained from each peak ID value. All statistical analyses were done with the vegan package of R (12).

**RNA isolation**

Overnight cultures of *S. meliloti*, grown in M9-succinate medium at 30˚C at 130 rpm, were diluted to an OD_600_ of 0.05 in 5 ml of M9-succinate and incubated until an OD_600_ of 0.4 was reached. Then, either 10 µM of luteolin (Sigma-Aldrich) or one of the alfalfa root exudate (normalized by the total organic carbon as measured by the CHNS analysis; 0.250 ml, 0.042 ml, 0.224 ml, and 0.806 ml for Camporegio, Lodi, Verbena, and the blank samples, respectively) was added to each of the cultures and incubated for an additional 4 hours at 30˚C with shaking at 130 rpm. Biological replicates were performed for each of the three strains across the five conditions. After incubation, cells were blocked with RNAprotect Bacteria (Qiagen, Venlo, The Netherlands) and total RNA was extracted using RNeasy Mini kits (Qiagen) from 0.5 ml of culture following the manufacturer’s instructions, including on column DNase I treatment. After elution, a second DNase I (ThermoFisher, Waltham, Massachusetts, USA) treatment was performed. The absence of contaminant DNA was verified by qPCR on the *nodC* gene of *S. meliloti*. Quality and quantity of extracted RNA were checked by spectrophometric readings (NanoQuant plate, Infinite PRO 200, Tecan, Männedorf, Switzerland), fluorometric measurement (Qubit, ThermoFisher), and cartridge electrophoresis on a 2100 Bioanalyzer (Agilent RNA Nano kit 6000, Agilent Technology, Santa Clara, California, USA). All RNA samples gave RNA Integrity Number (RIN) values between 9 and 10.

**Reverse transcriptase qPCR**

Single stranded cDNA libraries were prepared from total RNA samples using SuperScript II reverse transcriptase (ThermoFisher) following the manufacturer’s instructions. qPCR was performed using a QuantStudio^TM^ 7 flex (Applied Biosystems, Foster City, California, USA) programmed with the following temperature profile: 2 min at 94°C, followed by 40 cycles composed of 15 s at 94°C, 15 s at 60°C, and 30 s at 72°C, with a final melting curve to check for product specificity. Technical triplicate were carried-out as described in (8). Gene *smc01804* (*rplM*), encoding the 50S ribosomal protein L13, was used as a housekeeping gene for normalization of the expression data. The list of primers used is reported in **Table S2**. Relative quantification (RQ, as 2-ΔΔϹt) values were calculated with the ExpressionSuite ver. 1.0.4 software (Applied Biosystems). Differences on RQ data were evaluated by one-way ANOVA with Tukey pairwise contrast in R (9).

**RNA-sequencing library preparation**

Ribosomal RNA depletion was performed using MICROBexpress kits (ThermoFisher) following the manufacturer’s instruction starting from 0.6-1 µg of total RNA per sample. Removal of rRNA was checked on a Bioanalyzer 2100 (Agilent RNA Nano kit 6000, Agilent Technology). Ribosomal RNA depleted RNA preparations were used for library construction with TruSeq Stranded Total RNA Library Prep Gold kit (Illumina, San Diego, California, USA), using SuperScript II reverse transcriptase (ThermoFisher) for cDNA preparation. Libraries were assessed for quality using a DNA 1000 chip on a Bioanalyzer 2100 (Agilent Technologies), running 1 µl of each undiluted DNA library. Library normalization was performed based on Qubit fluorometric quantification. Libraries were sequenced on an Illumina Novaseq 6000 apparatus with a SP flow cell.

**Bioinformatic and statistical methods**

Reads were demultiplexed using “bcl2fastq2” version 2.2 with default parameters. Demultiplexed sequences were then quality controlled using the StreamingTrim algorithm (version 1.0) (13) with a quality threshold of 20 Phred. Quantification was done by Salmon (version 1.1.0) (14) against *decoy-aware* datasets containing both cDNA and the genome of each strain (as described in Salmon documentation: <https://salmon.readthedocs.io/en/latest/salmon.html#preparing-transcriptome-indices-mapping-based-mode>).

Heatmaps were prepared for each strain using the *ComplexHeatmap* and *Heatmaply* packages of R (15, 16).

Fold change values for all of the core *S. meliloti* genes were extracted and used to run a PCA using the *prcomp* function of R that was visualized with the *ggplot2* package of R (17). The same approach was used to compare expression of core genes across Rm1021, BL225C, and the hybrid strain.

For the Kyoto Encyclopedia of Genes and Genomes (KEGG) module annotations for each gene were extracted; if a gene was annotated with multiple KEGG modules, only the first one was kept. Then, for each strain-condition pairing, KEGG modules that were over- or under-represented among the up- and down-regulated genes (relative to the whole genome) were identified using hypergeometic tests (*p*-value < 0.05) with a custom R script. The same procedure was used to identify Cluster of Orthologous Genes (COG) categories that were over- or under-represented among the up- and down-regulated genes. All data processing was performed using custom Python scripts using Python ver. 3.6.9 and the external libraries Pandas ver. 0.23.4 (18), pickle ver. 4.0, and numpy ver. 1.15.4 (19). Hierarchical clustering was performed with the function “hclust” in R (9) on the log2 fold change of the statistically significant differentially expressed genes. UpsetR (20) was used to visualise intersecting dataset for KEGGs and COGs.

**References**

1. Meade HM, Long SR, Ruvkun GB, Brown SE, Ausubel FM. 1982. Physical and genetic characterization of symbiotic and auxotrophic mutants of Rhizobium meliloti induced by transposon Tn5 mutagenesis. J Bacteriol 149:114 LP – 122.

2. Carelli M, Gnocchi S, Fancelli S, Mengoni A, Paffetti D, Scotti C, Bazzicalupo M. 2000. Genetic diversity and dynamics of Sinorhizobium meliloti populations nodulating different alfalfa cultivars in Italian soils. Appl Environ Microbiol 66:4785–4789.

3. Galardini M, Mengoni A, Brilli M, Pini F, Fioravanti A, Lucas S, Lapidus A, Cheng J, Goodwin L, Pitluck S, Land M, Hauser L, Woike T, Mikhailova N, Ivanova N, Daligault H, Bruce D, Detter C, Tapia R, Han C, Teshima H, Mocali S, Bazzicalupo M, Biondi EG. 2011. Exploring the symbiotic pangenome of the nitrogen-fixing bacterium Sinorhizobium meliloti. BMC Genomics 12:235.

4. Meade HM, Signer ER. 1977. Genetic mapping of Rhizobium meliloti. Proc Natl Acad Sci 74:2076–2078.

5. Checcucci A, DiCenzo GC, Ghini V, Bazzicalupo M, Becker A, Decorosi F, Döhlemann J, Fagorzi C, Finan TM, Fondi M, Luchinat C, Turano P, Vignolini T, Viti C, Mengoni A. 2018. Creation and Characterization of a Genomically Hybrid Strain in the Nitrogen-Fixing Symbiotic Bacterium Sinorhizobium meliloti. ACS Synth Biol 7:2365–2378.

6. Teuber LR, Taggard KL, Gibbs LK, McCaslin MH, Peterson MA, Barnes DK. 1998. Fall dormancyStandard tests to characterize alfalfa cultivars’.(Ed. CC Fox) p. A-1.(North American Alfalfa Improvement Conference: Beltsville, MD).

7. Trabelsi D, Pini F, Aouani ME, Bazzicalupo M, Mengoni A. 2009. Development of real-time PCR assay for detection and quantification of Sinorhizobium meliloti in soil and plant tissue. Lett Appl Microbiol 48:355–361.

8. Checcucci A, Azzarello E, Bazzicalupo M, Galardini M, Lagomarsino A, Mancuso S, Marti L, Marzano MC, Mocali S, Squartini A, Zanardo M, Mengoni A. 2016. Mixed nodule infection in Sinorhizobium meliloti-medicago sativa symbiosis suggest the presence of cheating behavior. Front Plant Sci 7.

9. R Development Core Team. 2012. R: A language and environment for statistical computing. R Found Stat Comput Vienna, Austria.

10. Checcucci A, Azzarello E, Bazzicalupo M, Carlo AD, Emiliani G, Mancuso S, Spini G, Viti C, Mengoni A. 2017. Role and regulation of ACC deaminase gene in Sinorhizobium melilotr: Is it a symbiotic, rhizospheric or endophytic gene? Front Genet 8.

11. Giavalisco P, Köhl K, Hummel J, Seiwert B, Willmitzer L. 2009. 13C isotope-labeled metabolomes allowing for improved compound annotation and relative quantification in liquid chromatography-mass spectrometry-based metabolomic research. Anal Chem 81:6546–6551.

12. Oksanen J, Blanchet F, Kindt R, Legendre P, Minchin P, O’Hara R, Simpson G, Solymos P, Stevens M, Wagner H. 2013. vegan: Community Ecology Package. R package version 2.0-10. R Packag version.

13. Bacci G, Bazzicalupo M, Benedetti A, Mengoni A. 2014. StreamingTrim 1.0: A Java software for dynamic trimming of 16S rRNA sequence data from metagenetic studies. Mol Ecol Resour 14.

14. Patro R, Duggal G, Love MI, Irizarry RA, Kingsford C. 2017. Salmon provides fast and bias-aware quantification of transcript expression. Nat Methods 14:417–419.

15. Gu Z, Eils R, Schlesner M. 2016. Complex heatmaps reveal patterns and correlations in multidimensional genomic data. Bioinformatics 32:2847–2849.

16. Galili T, O’Callaghan A, Sidi J, Sievert C. 2018. heatmaply: an R package for creating interactive cluster heatmaps for online publishing. Bioinformatics 34:1600–1602.

17. Wickham Hadley. 2009. ggplot2: Elegant Graphics for Data Analysis. Springer-Verlag New York.

18. Reback J, McKinney W, jbrockmendel, Bossche J Van den, Augspurger T, Cloud P, gfyoung, Sinhrks, Klein A, Roeschke M, Hawkins S, Tratner J, She C, Ayd W, Petersen T, Garcia M, Schendel J, Hayden A, MomIsBestFriend, Jancauskas V, Battiston P, Seabold S, chris-b1, h-vetinari, Hoyer S, Overmeire W, alimcmaster1, Dong K, Whelan C, Mehyar M. 2020. pandas-dev/pandas: Pandas 1.0.3 https://doi.org/10.5281/ZENODO.3715232.

19. Oliphant TE. 2006. A guide to NumPy. Trelgol Publishing USA.

20. Conway JR, Lex A, Gehlenborg N. 2017. UpSetR: An R package for the visualization of intersecting sets and their properties. Bioinformatics 33:2938–2940.
