# Supplementary material for: Nonadditive Transcriptomic Signatures of Genotype-by-Genotype Interactions during the Initiation of Plant-Rhizobium Symbiosis
Source: mSystems. 2021 Jan 12;6(1):e00974-20. doi: 10.1128/mSystems.00974-20 (PMC7901481; doi:10.1128/mSystems.00974-20)
Supplement: TABLE S4 [file mSystems.00974-20-st004.pdf]

Supplemental Table S4.

A)

**Strain**	**Host plant of original isolation**	**Genome sequence**	**Reference**
Rm1021	*M. sativa*	GCA_000006965.1	[1]
AK83* (DSM23914)	*M. falcata*	GCA_000147795.3	[2]
BL225C (DSM23913)	*M. sativa*	GCA_000147775.3	[2]

* AK83 strain is also present, as original specimen after initial isolation, in the culture collection of All-Russia Institute of Agri- cultural Microbiology (RIAM, St. Petersburg, Russia).

B)

**Name of the cultivar**	**Germplasm type**	**Fall dormancy**	**Producer of the material**
Camporegio	*Medicago x varia (M. sativa x M. falcata)*	3	CREA-FLC, Lodi, Italy
Verbena	*M. falcata*	4	CREA-FLC, Lodi, Italy
Lodi	*M. sativa*	6	CREA-FLC, Lodi, Italy

C)

**Gene name**	**Locus tag**	**Strain**	**Forward sequence (5’-3’)**	**Reverse sequence (5’-3’)**
rplM	SMc01804	Rm1021	AAGCGGCCTTCGATGATCTG	CTCCACCGGCAGAAGTACAC
nodA	Sma0869	Rm1021	ACCACCAGGAGCTCTCAGAA	TATCCCGACCGAGTCGTAAG
nodB	Sma0868	Rm1021	TGAGATTGTCGAGGCAAGTG	GGATCTGCCGACCAATGTAT
sinI	SMc00168	Rm1021	TCCCGAAATCTCCCAGGATC	ACCGTGACGATATGGCTGAT
emrB	SMc03167	Rm1021	AATGCGTCGGGACTCTACAA	GCTGCGTGAGGATAGTGTTG
fixO2	SMa0766	Rm1021	GTTTCAGTGGGGATCGAAGC	GAGGTTCGATCATGTGCTGC
	SinmeB_6271	BL225C	ACAATCTTTTCGTCGCAGCC	CGAACAAGATCCGCCACAAT
	SinmeB_4750	BL225C	TATGTCGTCGGCCAGATCC	GCCGGTATAGCTTCCCTTGA

	Sinme_4706	AK83	CGAAGAGAAGACCAAAGGCG	CTGGATCTGACCCTTTTCGC
	Sinme_6882	AK83	GTAATGTGACGACGCCCTTC	ACGTTCCATTTGTCGGCTTT

1 Meade, H.M. *et al.* (1982) Physical and genetic characterization of symbiotic and auxotrophic mutants of Rhizobium meliloti induced by transposon Tn5 mutagenesis. *J. Bacteriol.* 149, 114 LP – 122

2 Galardini, M. *et al.* (2011) Exploring the symbiotic pangenome of the nitrogen-fixing bacterium Sinorhizobium meliloti. *BMC Genomics* 12, 235
